# Combined B2M Knockout and Alginate Encapsulation Reduces Immune Rejection of iPSC-Derived Pancreatic Progenitors

**DOI:** 10.3390/cells15211942

**Published:** 2026-10-26

**Authors:** Polina S. Ermakova, Anna A. Barinova, Alexandra Y. Bogomolova, Arseniy L. Potapov, Polina M. Zharova, Maxim A. Baten’kin, Mariya Yu. Zakharina, Alexey N. Konev, Sergey A. Chesnokov, Maria M. Karabut, Vadim V. Elagin, Alexandra N. Bogomazova, Aleksandra V. Kashina, Alexandra V. Panova, Elena V. Zagaynova

**Affiliations:** 1 Federal State Budgetary Institution of Higher Education “ Privolzhsky Research Medical University” of the Ministry of Health of Russia, Nizhny Novgorod 603005, Russiazharovapolina5@gmail.com (P.M.Z.);; 2 Lopukhin Federal Research and Clinical Center of Physical-Chemical Medicine, Moscow 119435, Russiaabogomazova@rcpcm.ru (A.N.B.);; 3 Federal State Budgetary Institution of Science, G.A. Razuvaev Institute of Organometallic Chemistry Russian Academy of Sciences, Nizhny Novgorod 603950, Russia; 4 Institute of Gene Biology Russian Academy of Sciences, Moscow 119334, Russia; 5 Vavilov Institute of General Genetics, Russian Academy of Sciences, Moscow 119333, Russia

**Keywords:** B2M knockout, alginate encapsulation, IPSC-derived pancreatic progenitors, immune rejection, NK cell response, macrophage adhesion, T-cell activation, diabetes mellitus, cell replacement therapy, biocompatibility

## Abstract

Allogeneic cell replacement therapy for diabetes using induced pluripotent stem cell (iPSC)-derived pancreatic progenitors (PPs) is currently limited by immune rejection. Alginate encapsulation and the genetic ablation of HLA-I via β2-microglobulin (B2M) knockout represent distinct strategies to reduce immune recognition and to protect the transplanted cells. We generated encapsulated B2M-knockout pancreatic progenitors (IPS-∆B2M-PPs) and evaluated their immunogenicity in vitro using each of: peripheral blood mononuclear cell (PBMC) co-culture, natural killer cell (NK cell) degranulation, T-cell activation, and rat macrophage adhesion. In vivo studies were also performed to assess capsule biocompatibility and structural stability, following transplantation into rat omentum. While the B2M knockout did not compromise differentiation to pancreatic lineage, encapsulation largely prevented NK cell degranulation regardless of the encapsulated cell type, although T-cells did show residual activation after 7 days of co-culture with encapsulated progenitors, albeit significantly reduced in comparison with non-encapsulated controls. IPS-∆B2M-PP-containing capsules showed reduced macrophage adhesion in vitro compared to wild-type progenitors, while Interleukin-1β (IL-1β) secretion by the macrophages remained negligible. In vivo, all capsules formed a myofibroblastic zone without giant foreign-body cells; IPS-∆B2M-PP implants exhibited the thinnest fibrotic capsules and minimal lymphocytic infiltration. No capsule deformation was observed at 2 weeks or 1 month, indicating preserved structural stability. Although this characterizes both the local tissue response to the biomaterial and capsule stability, it does not establish immune evasion or reduced immunogenicity of the encapsulated cells in vivo. Overall, this study demonstrates the feasibility of combining B2M knockout with alginate encapsulation and suggests a potential additional protective role for B2M deletion under conditions of compromised capsule integrity that requires further investigation.

## 1. Introduction

Cell replacement therapy using induced pluripotent stem cell (IPSC)-derived pancreatic progenitors (PPs) offers a promising cure for diabetes mellitus [1]. However, allogeneic transplantation faces two major immune barriers: adaptive immunity (T-cells recognizing donor human leukocyte antigen (HLA) molecules), and innate immunity (natural killer (NK) cells attacking cells lacking self-HLA class I (HLA-I) surface peptides).

Cytotoxic CD8+ T-cells recognize foreign HLA class I molecules (HLA-A, -B, -C) on donor cells. However, even with HLA-matched donors, minor histocompatibility antigens can trigger rejection, necessitating chronic immunosuppression, and this can have significant side effects [2,3]. A direct strategy to evade T-cell recognition is knockout of the B2M gene, which encodes β2-microglobulin—an essential subunit for cell surface expression of all the HLA class I molecules. B2M-deficient cells therefore lack HLA class I presentation, making them invisible to the CD8+ T-cells [4]. Unfortunately, eliminating HLA class I creates the other problem: NK-cell-mediated rejection via the “missing-self” mechanism. NK cells use inhibitory killer immunoglobulin-like receptors (KIRs) to detect self-HLA class I. Cells without HLA class I are therefore recognized as abnormal and lysed. Moreover, IPSC-derived cells can upregulate activating ligands (MICA, PVR, NECTIN2) while losing inhibitory signals, further sensitizing them to NK attack [1,5]. This is particularly relevant for B2M-knockout cells, which are highly vulnerable to NK cells unless additional protective modifications are introduced (e.g., CD47 overexpression or HLA-C retention).

An alternative or complementary strategy is alginate encapsulation—using a biocompatible polymer that creates a physical barrier between the transplanted cells and the host’s immune cells. Encapsulation prevents direct contact with T-cells, NK cells, and macrophages, while allowing diffusion of oxygen, nutrients, and insulin. Alginate is the most commonly chosen of several well-studied natural polysaccharides used for the encapsulation of biological objects. Alginate hydrogels exhibit good biocompatibility and are often used to create shells around pancreatic islets of Langerhans in order to produce stable transplants, making possible long-term normoglycemia, and to prevent fatal hypoglycemia in absolute insulin-deficient diseases. However, the clinical application of encapsulated cells is primarily limited by the so-called foreign body response (FBR)—a cascade of events leading to fibrotic overgrowth, impaired mass transfer, and eventual graft loss [6]. Furthermore, while encapsulation alone does not eliminate antigen shedding, or indirect immune recognition, the capsules themselves can cause inflammatory responses. Thus, combining encapsulation with genetic immune evasion may provide an additional layer of protection.

The combination of B2M knockout (to eliminate direct CD8+ T-cell recognition) and alginate encapsulation (to physically block access of immune effectors, including NK cells) represents a dual-mechanism strategy. While B2M-knockout cells remain susceptible to NK cells in vitro, encapsulation can prevent direct NK–target contact, potentially overcoming the missing-self vulnerability. This approach avoids the complexity of multi-gene editing (e.g., CD47 or HLA-C retention) while still addressing both arms of the immune system. Although B2M-knockout cells have been studied in non-encapsulated formats [4], and alginate encapsulation has been tested with wild-type cells [6], the combined efficacy of encapsulated B2M-knockout IPSC-derived pancreatic progenitors has not previously been evaluated in vitro in a controlled allogeneic co-culture system. Whether physical encapsulation can compensate for the NK cell susceptibility of B2M-deficient cells remains unknown.

Here we studied the in vitro possibility of overcoming immune reactions by generating encapsulated B2M-knockout IPSC-derived pancreatic progenitors, and testing their survival and immune evasion in vitro in co-culture with allogeneic human peripheral blood mononuclear cells (PBMCs). In vivo studies in rats were conducted to assess capsule biocompatibility and structural stability rather than graft immunogenicity or immune evasion following transplantation.

## 2. Materials and Methods

### 2.1. Cell Culture and In Vitro Differentiation of PSC Lines to Pancreatic Progenitor Cells

The IPSC lines RCPCMi007-A-1 and RCPCMi007-A (hereinafter referred to as IPS lines) were cultured as described previously [5]. The IPSC line with B2M knockout (IPS-∆B2M) was obtained earlier [7].

The thawed IPS and IPS-∆B2M cells were seeded onto six-well plates pre-coated with Matrigel (Corning, New York, NY, USA) in mTeSR1 medium (STEMCELL Technologies, Vancouver, BC, Canada) and cultured at 37 °C with 5% CO_2_ until at approximately 70% confluence. For differentiation, we used the STEMdiff™ Pancreatic Progenitor Kit (STEMCELL Technologies, Canada) according to the manufacturer’s instructions, with one modification: the Definitive Endoderm (Stage 1) was prolonged for 2 days instead of the standard protocol. Briefly, cells at 80–90% confluence were dissociated into single cells using Versene (Paneco, Gorki Leninskiye, Russia), and 2.6 × 10^6^ cells/cm^2^ (approximately 2.5 × 10^6^ cells per well of a 6-well plate) were seeded in mTeSR1 containing 10 µM Y-27632. Differentiation proceeded through Stages 1–4 as per the kit protocol. Upon completion of Stage 4, the medium was replaced with Stage 5 medium containing B27 (PanEco, Gorki Leninskiye, Moscow Region, Russia), glutamine (PanEco, Russia), 1 µM T3, 10 µM Alk5i (Abcam, Cambridge, UK), 10 µM zinc sulfate, and 10 µg/mL heparin (Sigma-Aldrich, St. Louis, MO, USA) for further maturation.

Fibroblasts were cultured in DMEM (PanEco) containing 10% FBS (ExCell, Shanghai, China).

### 2.2. DNA, RNA Extraction

The genomic DNA was extracted with the Extract DNA Blood Kit (Evrogen, Moscow, Russia). PCR was performed using the ScreenMix PCR amplification mix (Evrogen, Moscow, Russia). RNA was extracted with an Extract RNA kit (Evrogen, Moscow, Russia).

### 2.3. Real-Time PCR

cDNA synthesis was performed using a MMLV RT-kit (Evrogen, Russia). qPCR was performed using qPCRmix-HS SYBR (Evrogen, Russia) with gene-specific primers (Table S1) in a CFX96 Touch Real-Time PCR Detection System (Bio-Rad, Hercules, CA, USA). mRNA expression was normalized to the housekeeping gene RPL13A.

### 2.4. Immunocytochemistry

The pancreatic markers were assessed by immunofluorescence staining. Cells were fixed in 4% methanol-free paraformaldehyde (Thermo Fisher Scientific, Waltham, MA, USA), washed with phosphate-buffer saline (PBS) (Thermo Fisher Scientific), permeabilized with 0.5% Triton-X100, and blocked in a solution consisting of PBS, 5% Fetal Bovine Serum (FBS), 5% goat serum, and 0.1% Tween-20 (Applichem, Darmstadt, Germany) for 30 min. After overnight incubation at 4 °C with primary antibodies specific to PDX1 (Rabbit, 1:1000; Abcam, United Kingdom), glucagon (Mouse, 1:1000; Sigma-Aldrich, St. Louis, MO, USA), MafA (Rabbit, 1:200; Abcam, United Kingdom), the cells were washed and incubated with the secondary antibodies Alexa Fluor Plus 555 (Goat, anti-Mouse, 1:800) and Alexa Fluor 488 (Goat, anti-Rabbit, 1:800) (ThermoFisher, USA) for 1 h at room temperature. Subsequently, the cells were washed and incubated with Alexa Fluor 647 Mouse anti-NKX6.1 (Mouse, 1:20; BD Pharmingen, San Diego, CA, USA).

For nuclear staining, 400 ng/mL DAPI (Sigma-Aldrich, USA) was used. Staining was performed for 15 min, followed by three washes with PBS. Cells were visualized under an Olympus FV3000 (Tokyo, Japan) confocal microscope.

### 2.5. Flow Cytometry

Cells were removed from the substrate with a 0.05% trypsin solution and incubated with the enzyme for up to 10 min at room temperature. The enzyme was inactivated with DMEM/F12 medium (PanEco) with 10% FBS, after which the cell suspension was centrifuged for 5 min at 300× *g*. The cells were resuspended in PBS solution without calcium or magnesium ions, and 200,000 cells were selected in a separate eppendorf tube for use as an autofluorescence control. Then, 5 µL/test of PE anti-human CD184 (CXCR4) antibodies (BioLegend, San Diego, CA, USA) was added to the cells in the control tubes, which were then incubated in the dark for 25 min, before adding 500 µL of PBS and centrifuging at 300× *g* for 5 min. The cells were analyzed on an Acea NovoCyte 3000 flow cytometer (ACEA Biosciences, San Diego, CA, USA).

The pancreatic markers were assessed by immunofluorescence staining. Cells were removed from the substrate with a 0.05% trypsin solution by incubating them with the enzyme for up to 10 min at room temperature. The enzyme was then inactivated with DMEM/F12 medium (PanEco) containing 10% FBS, after which the cell suspension was centrifuged for 5 min at 300× *g*, as was also done in subsequent stages. The cells were washed twice with cold PBS without calcium or magnesium ions; at this and the following stages, centrifugation was carried out at 4 °C. When selecting the supernatant, 100 μL of the solution was left in the tube and the cells were carefully resuspended in this, after which 1 mL of 1% PFA was added before incubation on ice for 15 min. The cells were washed twice with cold PBS without calcium or magnesium ions, followed by centrifugation. When selecting the supernatant, 100 μL of the solution was left, the cells were carefully resuspended in it, and 1 mL of 80% ice-cold ethanol was added before incubation on ice for 30 min. Then, 0.5 mL of cold PBS without calcium or magnesium ions was added, and the cells were centrifuged. After that, the PBS cells were washed twice without calcium or magnesium ions; before the second washing, each sample was divided in half, and 200,000 cells were selected in a separate eppendorf for use as an autofluorescence control. The experimental cells were resuspended in a pre-prepared cold PBS solution without calcium or magnesium ions but with the addition of 1% FBS containing antibodies to Alexa Fluor 647 Mouse anti-NKX6.1 (BD Pharmigen, USA) and PE Mouse anti-PDX1 (BD Pharmigen, USA) 1:20 and incubated on ice for 1 h. The cells were washed twice with cold PBS without calcium or magnesium ions but with the addition of 1% FBS, and 300,000 cells were resuspended in 140 µL of a DAPI solution with a concentration of 100 ng/mL.

On day 16 of cell differentiation, the levels of HLA class I molecules (HLA-A, B, C) were assessed. The cells were stained for mouse monoclonal anti-human HLA-A,B,C antibody (clone w6/32), (Sony Biotechnology, San Jose, CA, USA). The antibodies were added at a concentration of 1.5 µL per 100 µL of cell suspension. Staining was performed for 30 min at 4 °C in the dark, followed by washing with PBS buffer. The HEK293 cell line was used as a control. The cells were analyzed on an Acea NovoCyte 3000 flow cytometer (ACEA Biosciences, San Jose, CA, USA).

### 2.6. Capsule Synthesis

To provide physical immunoprotection for transplanted pancreatic progenitors, we developed alginate-based microcapsules using a custom microfluidic device for droplet formation. In this device, the dispersed phase initially forms alginate microspheres, which are then crosslinked in the gelling solution. These microspheres are subsequently coated with a semipermeable PMETAC:NVP membrane and an additional thin alginate layer, resulting in a core–shell architecture. The microfluidic encapsulation setup and its operational parameters are protected as a trade secret under Order No. 249/Akd (dated 9 February 2022). Additionally, the overall encapsulation methodology and device architecture were implemented according to a proprietary design protected under Patent RU2822875C1. The capsules were prepared according to the protocol described previously [1,8,9].

Prior to use, the microfluidic device was sterilized by flushing with 70% ethanol and exposure to ultraviolet (UV) irradiation for 15 min. All removable and accessory components were sterilized by autoclaving. All solutions were adjusted to an isotonic osmotic pressure of 0.75 Mpa and their pH was adjusted to 7.4 using sodium hydroxide. The sodium alginate and polyethylene glycol solutions were sterilized by autoclaving. Before use, both the washing buffer and gelling solution were filtered through polyethersulfone membrane filters with a pore size of 0.22 µm.

For capsule synthesis, very low viscosity sodium alginate was used (viscosity <20 mPa·s at 1% (20 °C), with a guluronate (G) content ≥60%, PRONOVA^®^ UP VLVG, NovaMatrix, Sandvika, Norway). For the preparation of empty microcapsules, the dispersed phase (capsule core material) consisted of 2 wt% sodium alginate (PRONOVA^®^ UP VLVG), 15 wt% dextran (Mw 20,000 Da, AO “Vector”, Novosibirsk, Russia), 10 mmol/L 3-(N-morpholino)propanesulfonic acid (MOPS, Cat. No. M3183, Sigma-Aldrich, St. Louis, MO, USA), and 0.53 wt% sodium chloride (NaCl, AO “Vector”, Saint Petersburg, Russia). For the preparation of cell-containing microcapsules, the alginate composition was adjusted to 2.7 wt% sodium alginate, 20 wt% dextran, 13.3 mmol/L MOPS, and 0.41 wt% NaCl. A suspension of pancreatic cells in culture medium was added to the alginate composition at a 1:3 volume ratio immediately prior to encapsulation. To visualise the microcapsule formation process, the dispersed phase was supplemented with methylene blue (Khimreaktiv, Nizhny Novgorod, Russia) as a dye. The continuous phase consisted of 30 wt% polyethylene glycol (Mw 8000 Da, Nizhnekamskneftekhim, Nizhnekamsk, Russia), 10 mmol/L MOPS, and 0.73 wt% NaCl.

The resulting microdroplets were crosslinked in a gelling solution containing 2.7 wt% barium chloride dihydrate (BaCl_2_·2H_2_O, Khimreaktiv, Russia) and 10 mmol/L MOPS. After crosslinking, and after each subsequent step, the microcapsules were washed in a washing buffer consisting of 10 mmol/L MOPS and 0.84 wt% NaCl. Subsequently, a semipermeable polymer membrane was formed on the surfaces of the microcapsules by incubation for 10 min in a solution of the cationic copolymer of [2-(methacryloyloxy)ethyl]trimethylammonium chloride (METAC, 80 wt% solution in water, Cat. No. 408107, Sigma-Aldrich, St. Louis, MO, USA) and N-vinylpyrrolidone (NVP, ≥99%, Sigma-Aldrich, St. Louis, MO, USA) at a 1:2 molar ratio (PMETAC:NVP, 1:2) at a concentration of 0.5%. After polymer coating, an additional alginate layer was applied to the surface of the polymer membrane by incubating the microcapsules in 0.05 wt% sodium alginate solution in a buffer for 5 min. The resulting capsules consisted of an alginate hydrogel core covered by a semipermeable PMETAC:NVP copolymer membrane, which was in turn coated with a thin alginate shell.

### 2.7. Microcapsule Stability Under Physiologically Relevant Conditions

The stability of the microcapsules was evaluated under conditions mimicking the physiological environment to ensure their suitability for transplantation.

The microcapsules were incubated in three different media at 37 °C: Hank’s Balanced Salt Solution (HBSS), complete culture medium (DMEM with 10% FBS containing an antibiotic and antimycotic agents), and fetal bovine serum. At 1 h and at 24 h post-incubation, the microcapsules were examined using bright-field microscopy (Leica DM2500, Berlin, Germany), and their diameters were measured using ImageJ 1.43u software. Their stability was assessed by monitoring changes in microcapsule diameter and morphological integrity, including the presence of deformation, shrinkage, or loss of optical contrast.

### 2.8. Permeability Measurements

The permeation test was carried out at 4 °C. The microcapsules were incubated for 48 h at 4 °C in solutions containing FITC-labeled lectins: (10 mg/µL) FITC-Triticum Vulgare (WGA, molecular weight 36 kDa), 14 µL (2 mg/µL) FITC-Maackia amurensis I (MAL-I, molecular weight 75 kDa), 5 µL (10 mg/µL) FITC Ricinus communis (RCA-I, molecular weight 120 kDa), and FITC-Sambuca nigra (SNA, molecular weight 150 kDa).

Following incubation with the lectins, the microcapsules were examined using confocal microscopy (LSM 880, Carl Zeiss, Mainz, Germany). Their permeability was quantified by calculating the ratio of mean fluorescence intensity inside the capsules (in the region of the encapsulated cells) to that outside the capsules using ImageJ software. A ratio ≤ 1 was considered indicative of impermeability, demonstrating an effective barrier against the corresponding molecule.

### 2.9. Viability

To evaluate the impact of the encapsulation process and its constituent solutions on cell viability, a Live/Dead cell double-staining assay was performed using the Live/Dead Cell Double Staining Kit (Sigma-Aldrich, Saint Louis, MO, USA), according to the manufacturer’s instructions.

Viability was assessed exclusively at 24 h post-encapsulation using the same Live/Dead double-staining protocol described above. Due to the three-dimensional architecture of the capsules, we used confocal fluorescence microscopy with optical sectioning. Both individually embedded cells and small cell clusters within the capsules were analyzed. The percentage of viable cells was calculated by measuring the green-stained (live) area relative to the total cell-stained area (live + dead) using ImageJ software, as described previously. This approach enabled semi-quantitative evaluation of regional viability differences within the encapsulated constructs. All experiments were performed in triplicate, and the data are presented as means ± standard deviation (SD).

### 2.10. Immune Tests—Incubation with Rat Macrophages

Rat peritoneal macrophages were isolated using a rapid peritoneal lavage method to dislodge the resident macrophages from the peritoneal surface. The peritoneal cells were plated into 24-well plates with RPMI-1640 medium supplemented with L-glutamine, 10% heat-inactivated fetal bovine serum (hiFBS), and a 1% antibiotic–antimycotic solution. After 12 h of incubation (37 °C, 5% CO_2_), the culture medium was aspirated, and the adherent monolayer was gently rinsed with 0.5 mL of PBS to remove non-adherent cells. Subsequently, 100 microcapsules were added to each well and co-incubated with the adherent macrophages for an additional 24 h.

For visualization of adherent cells, any macrophages attached to the microcapsule surfaces were stained using the Live/Dead fluorescence viability kit and examined under a fluorescence microscope (EVOS M7000) and a confocal laser scanning microscope (LSM 880, Carl Zeiss, Mainz, Germany). Quantitative assessment of adhesion was performed by counting the number of macrophages attached to each individual microcapsule. In each experimental group, at least 50 randomly selected microcapsules were analyzed, and the results were expressed as the mean number of cells per microcapsule.

Macrophage activation was evaluated by measuring the secretion of the pro-inflammatory cytokine interleukin-1β (IL-1β) after 24 h of co-incubation. IL-1β concentrations in the culture supernatants were determined using an ELISA kit (Cloud-Clone Corp., Wuhan, China). Macrophages stimulated with bacterial lipopolysaccharide (LPS, 100 ng/mL; Servicebio, Wuhan, China) served as the positive control, while macrophages cultured in medium without microcapsules or LPS were used as the negative control.

### 2.11. Immune Responses of Human and NK Cells

To evaluate the NK cell and T-cell immune responses to pancreatic progenitors, peripheral blood mononuclear cells (PBMCs) were isolated from six healthy donors. The PBMCs were obtained by density gradient centrifugation using Ficoll (PanEco, Russia, Cat. No. P050E). Three days before the assay, adherent target cells (fibroblasts, IPS-PPs, and IPS-∆B2M-PPs) were seeded onto 96-well plates in triplicate for each donor at a density of 10,000 cells/cm^2^ in their respective growth media. One day before the experiment, the PBMCs were resuspended in RPMI-1640 medium (PanEco, Russia) supplemented with 10% heat-inactivated FBS (Gibco, Thermo Fisher Scientific: Waltham, MA, USA) and 500 IU/mL IL-2 (SCAN BIOTEK, Redkino, Tver Region, Russia) at a density of 1 × 10^6^ cells/mL. On the day of the experiment, the PBMCs were resuspended in RPMI-1640 medium (PanEco, Russia) containing 10% FBS (Gibco, USA), antibodies against CD56 (1:1000, Sony Biotechnology, Tokyo, Japan, Cat. 2191530), and antibodies against CD107a (1:1000, Sony Biotechnology, Japan, Cat. 2191530). Empty capsules, as well as capsules containing fibroblasts or pancreatic progenitors, were resuspended in 60 µL of the PBMC suspension with antibodies and transferred to 96-well plates in triplicate at 25–35 capsules per well. The K562 cell line was used as a positive control and plated at 40,000 cells per well. For adherent cultures, the growth medium was replaced with 60 µL of the PBMC suspension containing antibodies. The incubation was carried out for 4 h. The cells were then washed to remove unbound antibodies, and fluorescence was assessed using a Novocyte 3000 flow cytometer (ACEA Biosciences, San Diego, CA, USA). The obtained data were normalized for each donor to the mean percentage of degranulated NK cells in co-culture with K562, which was set as the maximum degranulation level (100%). Accordingly, the degranulation index was calculated as a percentage of this positive-control value. Experiments were performed using three independent differentiations into pancreatic progenitors, with two independent encapsulations per differentiation. Each condition was tested on two PBMC donors in three technical replicates. The gating strategies for the flow cytometry analysis for immune tests with human cells are shown in Figures S1–S3.

### 2.12. Immune Response of Human T-Cells

To assess the T-cell immune response, two stimulation protocols were used: a 1-day (early activation), and a 7-day (late activation) protocol. For the 1-day protocol, PBMCs from 2 healthy donors were used, and for the 7-day protocol, PBMCs from a total of 4 healthy donors were used. In both cases, adherent target cells (fibroblasts, IPS-PPs, and IPS-∆B2M-PPs) were seeded onto 48-well plates in triplicate at a density of 10,000 cells/cm^2^ in their respective growth media three days before the experiment. On the day of the experiment, PBMCs without prior stimulation were mixed with the targets at 300,000 cells per well in EX-VIVO^®^15 medium (Lonza Bioscience, Basel, Switzerland) supplemented with 10% FBS (Gibco, USA) and 50 IU/mL IL-2 (SCAN BIOTEK, Russia). Capsules were used at 40–60 per well. After 24 h, the cells were washed and stained with antibodies against CD3-APC (1:800, Sony Biotechnology, Japan), CD25-PE (1:800, Sony Biotechnology, Japan), and CD69-FITC (1:800, Sony Biotechnology, Japan). Cells positive for all three markers were considered activated. Data were normalized to the percentage of activated cells in the unstimulated control. For the 7-day stimulation, the protocol was identical except for the staining: CD3 and CD69 antibodies were used for the flow cytometry. The 7-day experiments were performed using 2 differentiations into pancreatic progenitors, with two independent encapsulations per differentiation, with each data point assessed in three technical replicates, whereas the 1-day experiment used only one differentiation with two encapsulations in triplicate. The gating strategies for the flow cytometry analysis for immune tests with human cells are shown in Figures S1–S3.

### 2.13. In Vivo Biocompatibility

To evaluate the host tissue response to the microcapsules in vivo, microcapsules were transplanted into the omentum of rats. The study used Wistar rats aged 1–2 months. All animal procedures were approved by the Bioethics Committee of the Privolzhsky Research Medical University (Protocol No. 13 dated 18 June 2024) and performed in accordance with the relevant national and institutional guidelines for the care and use of laboratory animals.

The study included four experimental groups based on the type of graft content transplanted into the omentum: microcapsules containing IPS-PP, microcapsules containing IPS-ΔB2M-PP cells, microcapsules containing primary fibroblasts (serving as a cellular positive control), and empty microcapsules (serving as a negative control to assess the host response against the capsule material alone). Prior to surgery, the animals were anesthetized with Zoletil (Virbac, Hamilton, New Zealand) at 20 mg/kg and xylazine (Bayer, Leverkusen, Germany) at 6 mg/kg. A small midline laparotomy was made, and the omental tissue was exteriorized and spread onto moist gauze. A total of 2000 microcapsules suspended in 1 mL of sterile physiological saline were transplanted into the omentum using an 18G needle, following the technique previously described by Paret et al. [10] and adapted by Ermakova et al. [8]. Surgical access to the abdominal cavity was achieved through a 0.5–0.7 cm midline incision. After dissection of the skin and the underlying aponeurosis (linea alba), the omentum was carefully exteriorized. A total of 2000 microcapsules (approximately 1 mL) were suspended in 1 mL of sterile physiological saline (total volume 2 mL) and injected into the omentum using an 18G needle. To minimize capsule damage during injection, the microcapsule suspension was injected slowly and continuously through the wide-bore needle, and the omentum was kept moist during the procedure. The injection was performed under visual control, and the capsules were distributed evenly within the omental tissue. After transplantation, the omentum was returned to the abdominal cavity, and the incision was sutured. All animals received antibiotic therapy (Augmentin, 1 mg/mL) for 3–5 days postoperatively. No immunosuppressive drugs were administered at any time during the study.

Grafts were retrieved at two predefined time points: 2 weeks (early response), and 1 month (chronic response) post-transplantation (*n* = 3 per group at each time point). Explanted tissues were fixed in 10% neutral buffered formalin, dehydrated through a graded ethanol series, and embedded in paraffin.

For general histological evaluation, hematoxylin and eosin (H&E) staining was used, while Masson’s trichrome helped in assessing fibrosis, and immunohistochemical staining with anti-smooth muscle antibody (αSMA) and anti-macrophage cytoskeletal protein antibody (ionized calcium-binding adapter molecule 1, Iba-1) were used to assess the extent of localization around the microcapsules of the myofibrobroblasts and macrophages, respectively.

The histology slides were digitized using the EVOS M7000 all-in-one imaging system (Thermo Fisher Scientific) and quantified using QuPath 0.7.0 open source software. The thickness of the myofibroblastic zones, the thickness of the fibrotic zones around the microcapsules, the number of destroyed microcapsules, the density of blood capillaries and their distance from the microcapsules were quantitatively assessed.

### 2.14. Statistics

The statistical analyses were conducted using GraphPad Prism 9.0 software (GraphPad Software, La Jolla, CA, USA). The distribution of all data was first checked for normality using the Shapiro–Wilk test. Depending on the results of these tests, data are presented as the median ± 25th and 75th quartiles for non-parametrically distributed data, or as the mean ± standard deviation (SD) for parametrically distributed data. Most data are visualized as box plots, where the boxes represent the interquartile range, the lines inside the boxes indicate the median, and the whiskers show the minimum and maximum values.

For comparisons between any two independent groups, the non-parametric Mann–Whitney U test was used. Independent t-tests were applied for pairwise comparisons of parametrically distributed data. For comparisons involving more than two groups, normally distributed data were analyzed by one-way ANOVA followed by Dunnett’s T3 multiple comparisons test (used to account for unequal variances between groups), while non-normally distributed data were analyzed by the Kruskal–Wallis test followed by Dunn’s multiple comparisons test, in accordance with the Shapiro–Wilk test results. For all statistical tests, a *p*-value of less than 0.05 was considered statistically significant. In the figures, non-significant differences are indicated as “ns”, and the levels of significance are denoted as follows: *—*p* < 0.05; **—*p* < 0.01; ***—*p* < 0.001; and ****—*p* < 0.0001.

For each cell line, three independent batches of microcapsules were produced, each from an independent cell thawing followed by the application of each respective encapsulation procedure. All analyses were performed on these three batches, and the results were pooled. Specifically, microcapsule size was measured on 50 capsules per batch (150 capsules per condition across three batches); permeability and viability were each assessed on 10 capsules per batch (30 capsules per condition across three batches); macrophage adhesion was quantified on at least 50 capsules per batch (150 capsules per experimental group across three batches). IL-1β secretion was measured in culture supernatants from macrophages co-incubated with 100 microcapsules per well; one supernatant sample per batch was analyzed by ELISA (n = 3 independent batches, each tested in triplicate). Human NK- and T-cell assays were performed: using PBMCs from six healthy donors for the NK assay, from four for the 7-day T-cell assay, and from two with the 24 h-T-cell test. Each assay involved three independent capsule batches and three technical replicates per donor. In vivo experiments were performed using n = 3 animals per group per time point (2 weeks and 1 month), with three experimental groups (IPS-PP-loaded, IPS-ΔB2M-PP-loaded, and fibroblast-loaded capules), involving a total of 18 animals.

## 3. Results

### 3.1. Differentiation B2M-Knockout IPS Cells into Pancreatic Cells

**Figure 1:**
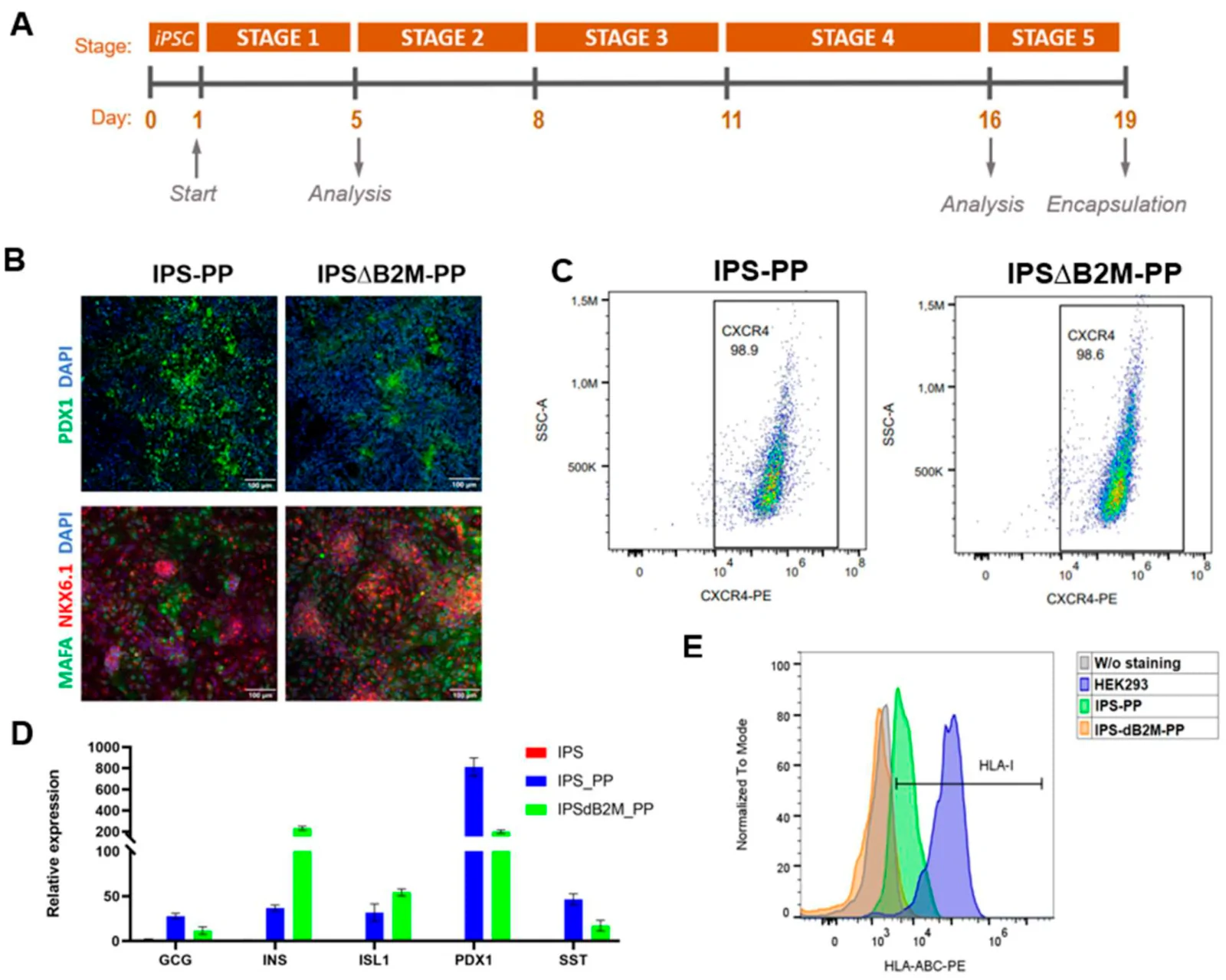
(**A**) Scheme of IPSC differentiation to pancreatic progenitor cells; (**B**) fluorescence microscopy of pancreatic progenitor cells derived from IPS cells and B2M-deleted IPS cells (left to right); staining for PDX1 (green) and GCG (red); scale bar: 100 µm. Fluorescence microscopy of pancreatic progenitor cells derived from IPS cells and B2M-deleted IPS cells (left to right); staining for MafA (green), NKX6.1 (red) and DAPI (blue); scale bar: 100 µm. (**C**) Flow cytometry analysis of CXCR4+ cells at the DE stage of differentiation. (**D**) mRNA expression levels of pancreatic progenitor markers (ISL1, PDX1) and hormones (GCG, INS, and SST). (**E**) Anti-HLA-A,B,C immunostaining at Day 16 of differentiation.

To overcome any possible immune rejection after cell replacement therapy for diabetes mellitus (DM), we chose the IPS line with decreased potential immunogenicity as a result of β2-microglobulin (B2M) disruption. Such B2M-knockout IPS cells had been generated and characterized previously [5]. Next, we differentiated unedited and edited cell lines (IPS and IPS-∆B2M, respectively), into pancreatic progenitor cells (IPS-PPs and IPS-∆B2M-PPs, respectively), by using the commercially available Pancreatic Progenitor Kit (Stem Cell Technologies, Vancouver, BC, Canada), with modifications (Figure 1A). The most crucial stage of early pancreatic differentiation is the Definitive Endoderm (DE) stage (Stage 1), so this was prolonged for 2 days, and at day 5 after the induction of differentiation, more than 98% of cells were positive for the surface DE marker CXCR4 (Figure 1C). At day 16 of differentiation, cells in 2D culture were analyzed for the pancreatic progenitor (PP) markers PDX1, NKX6.1 and MAFA (Figure 1B). To assess the efficiency of differentiation, both cell lines were analyzed for expression of pancreatic genes including the following hormones: beta-cell hormone (insulin), alpha-cell hormone (glucagon), and delta-cell hormone (somatostatin) (Figure 1D). Both cell lines had increased expression of specific pancreatic genes compared to undifferentiated IPSCs (Figure 1D). We did not observe any substantial differences in the percentage of PDX1, NKX6.1 and MAFA positive cells between the WT and B2M-knockout cells at this pancreatic progenitor induction stage.

### 3.2. HLA-I Expression in IPSC-Derived Pancreatic Progenitor Cells

We next analyzed whether 16 days of differentiation was enough to induce significant HLA-I expression in pancreatic progenitor cells. At day 16 of differentiation, 2D culture cells were collected by trypsinization and stained with anti-HLA-A,B,C antibodies (Figure 1E). The HEK293T cell line was used as a positive for HLA-I expression control. After 16 days of differentiation, the proportion of HLA-I-positive cells in the non-knockout IPS-PP cell line had increased to 40%, compared to the B2M knockout cells, IPS-∆B2M-PP, in which no antigen expression was observed.

### 3.3. Alginate Microcapsule Fabrication and Characterization

#### 3.3.1. Microcapsule Synthesis and Stability

**Figure 2:**
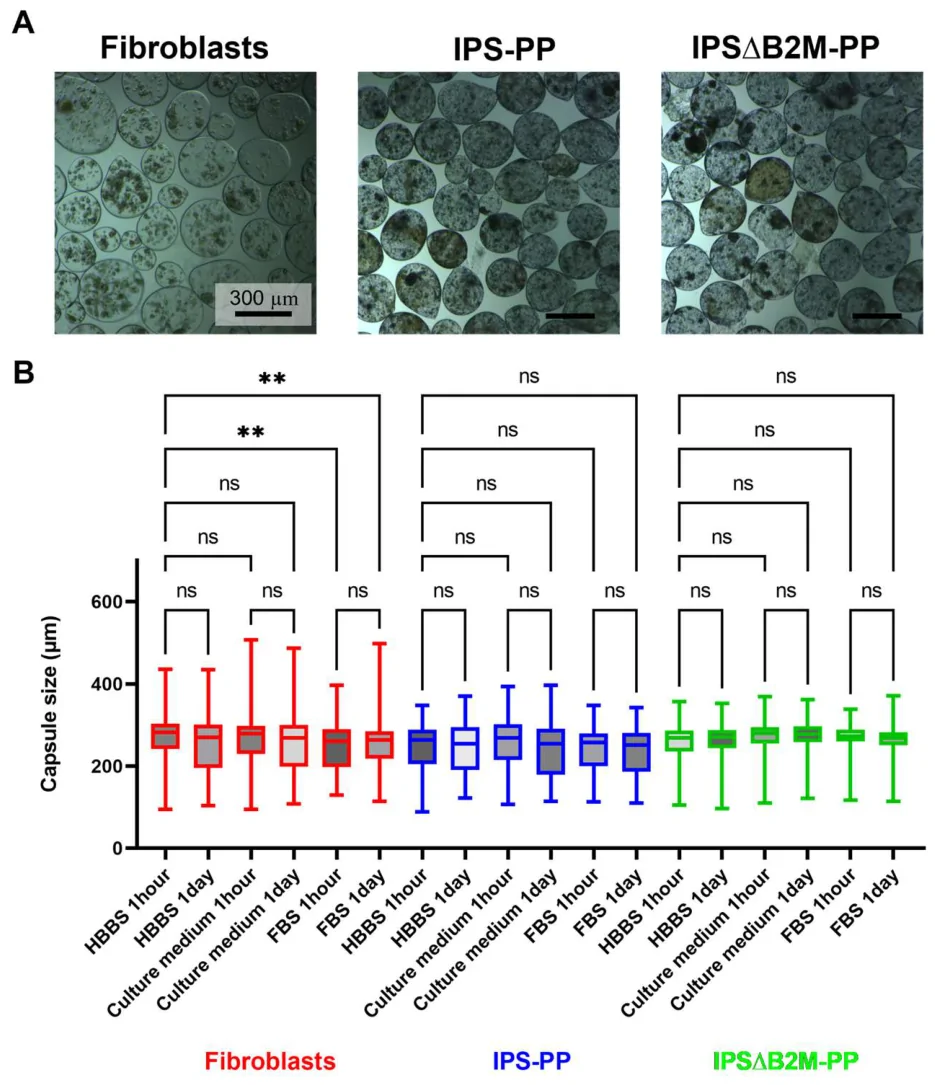
Characterization of alginate microcapsule morphology and stability. (**A**) Representative bright-field images of alginate microcapsules (PMETAC:NVP (1:2) coating) loaded with fibroblasts, IPSC-derived pancreatic progenitors (IPS-PPs), or B2M-knockout pancreatic progenitors (IPS-∆B2M-PPs). Scale bar, 300 μm. (**B**) Quantitative analysis of microcapsule size stability. Microcapsules were incubated for 24 h in Hank’s Balanced Salt Solution (HBSS), complete culture medium (DMEM with 10% FBS), or fetal bovine serum (FBS). Three independent batches with 50 capsules each (total = 150 capsules per group) were used. Statistical test: Kruskal–Wallis test followed by Dunn’s multiple comparisons test. Statistical significance: ** *p* < 0.01, ns—non-significant differences are indicated.

To provide physical immunoprotection for transplanted pancreatic progenitors, we developed alginate-based microcapsules using a custom microfluidic device for controlled droplet formation. The resulting capsules had a mean diameter of 235–270 μm. The polydispersity index (PDI) ranged from 0.034 to 0.070 (PDI < 0.1 indicating a narrow size distribution) across all cell types and incubation conditions (Table S2). Occasional outliers ranged from ~100 μm to 350–400 μm, but the overall size distributions were reproducible across independent syntheses. Notably, capsules containing fibroblasts appeared optically brighter than those containing IPS-PP or IPS-∆B2M-PP cells, possibly reflecting differences in residual culture medium entrained during encapsulation (Figure 2A).

To assess the reproducibility and physical integrity of the microcapsules for potential transplantation, we evaluated their stability under physiologically relevant conditions (HBSS, culture medium, and serum). The results show that all capsule types maintained their dimensions over 24 h, with no significant swelling or shrinkage (*p* > 0.05) (Figure 2B). Although fibroblast-loaded capsules exhibited a slight but observable size increase in serum-containing medium, while IPS-PP and IPS-∆B2M-PP capsules remained unchanged, this variation did not compromise capsule integrity or uniformity. These findings confirmed that, regardless of the encapsulated cell type, the microcapsules possessed adequate mechanical stability and size reproducibility for further in vitro and in vivo evaluation.

To further assess the mechanical resilience of the capsule shell, we performed an osmotic stability test based on exposure to a hypotonic solution, a validated model of mechanical loading that reveals latent defects in the capsule material [11,12]. Exposure to distilled water caused reversible elastic swelling of the capsules (319.9 ± 57.7 μm vs. 253.1 ± 68.7 μm in HBSS; *p* < 0.0001), followed by full recovery of size after return to HBSS, with no rupture or loss of integrity at any time point. These results indicate that the microcapsules possess sufficient elasticity and structural resilience to withstand osmotic stress without irreversible deformation (Supplementary Text S1, Figure S4).

#### 3.3.2. Permeability

To determine whether the microcapsules provide an effective immunoisolating barrier, their permeability was assessed using FITC-conjugated lectins of increasing molecular weights (36, 75, 120, and 150 kDa). Fluorescence intensity was measured inside the capsules in the region of the encapsulated cells, as lectins possess affinity for cell surface glycans and tend to bind to the cells.

Effective immunoisolation requires a membrane that permits diffusion of low-molecular-weight molecules such as glucose (180 Da) and insulin (5.8 kDa) while blocking immunoglobulins (~150 kDa) and complement components (~185 kDa) [13]. Accordingly, the 36 kDa lectin should freely penetrate, whereas the 120 kDa and 150 kDa lectins should be effectively excluded. The 75 kDa lectin, however, represents a borderline case. Some studies consider that capsules should be impermeable to 75 kDa lectin to ensure exclusion of pro-inflammatory cytokines such as TNF-α (51 kDa) [14]. By contrast, other studies consider permeability for lectins ≤ 40 kDa acceptable, demonstrating that capsules with a cut-off in this range remain impermeable to ≥150 kDa molecules while allowing sufficient nutrient and insulin diffusion [15]. Others have proposed an exclusion threshold of 78–103 kDa as sufficient for immunoisolation [16]. Thus, permeability for the 75 kDa lectin requires experimental evaluation and is not a definitive criterion for immunoisolation efficacy.

**Figure 3:**
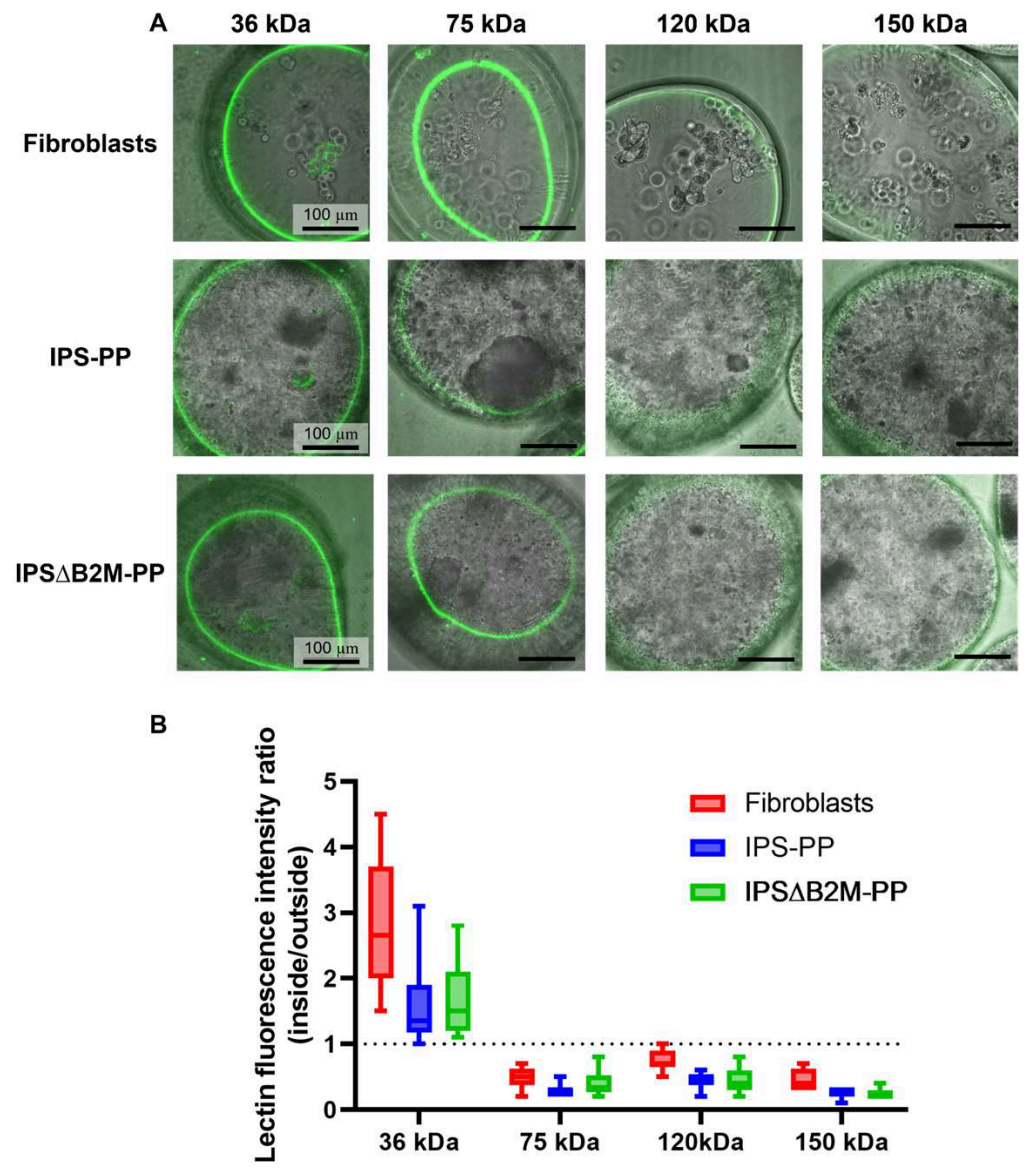
Permeability profile of microcapsules. (**A**) Representative confocal fluorescence images of microcapsules (loaded with fibroblasts, IPS-PPs, or IPS-∆B2M-PPs) after 48-h incubation with FITC-conjugated lectins of a range of molecular weights (36, 75, 120, and 150 kDa). Green fluorescence indicates lectin penetration. Scale bar, 100 μm. (**B**) Quantitative analysis of the outside/inside fluorescence intensity ratios. A ratio > 1 indicates free diffusion of the lectin into the capsule, whereas a ratio ≤ 1 indicates effective exclusion. Three independent batches with 10 capsules each (total = 30 capsules per group) were used.

Our experimental results demonstrated that, regardless of the encapsulated cell type, all capsules exhibited a consistent permeability profile (Figure 3A,B). As expected, the 36 kDa lectin readily penetrated the capsules, with outside/inside fluorescence ratios of 1.5–2.8, confirming the free diffusion of low-molecular-weight molecules. In contrast, penetration by the 120 kDa and 150 kDa lectins was effectively blocked, with ratios consistently below 1.0 (ranging from 0.2 to 0.8), confirming that the capsules provide an effective barrier against high-molecular-weight immune components. Collectively, our data establish an apparent permeability threshold between 75 and 120 kDa, indicating size-selective permeability of the capsule membrane. This profile is consistent with the requirements for immunoisolation, as it permits diffusion of low-molecular-weight molecules while restricting the passage of high-molecular-weight immune effectors.

#### 3.3.3. Cell Viability Assessment

**Figure 4:**
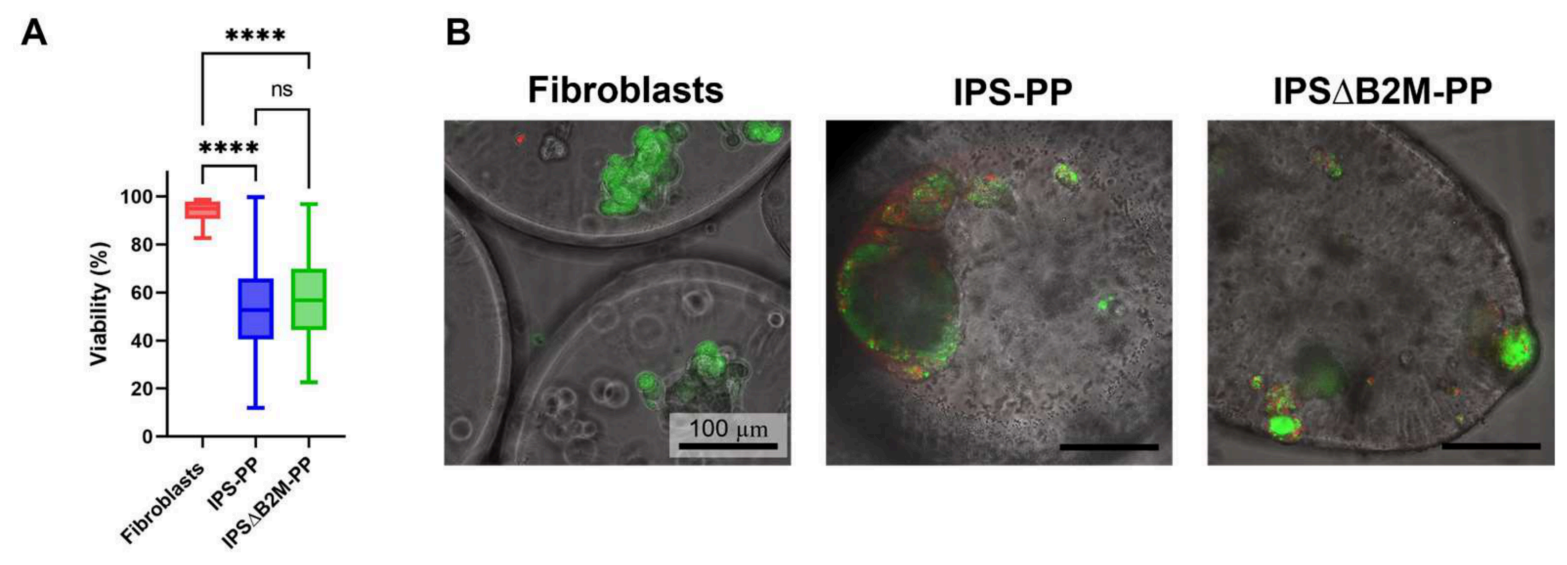
(**A**) Viability of encapsulated cells 24 h post-encapsulation. (**B**) Representative confocal fluorescence images of alginate microcapsules loaded with fibroblasts, IPS-PPs, or IPS-∆B2M-PPs, stained with Live/Dead assay (green—live cells, red—dead cells). Three independent batches with 10 capsules each (total = 30 capsules per group) were used. Statistical test: Kruskal–Wallis test followed by Dunn’s multiple comparisons test. Statistical significance: **** *p* < 0.0001, ns—non-significant differences are indicated.

To evaluate whether the encapsulation process adversely affects cell survival, viability was assessed 24 h post-encapsulation. Fibroblasts demonstrated high viability, with mean values of 93.4 ± 5.3%. In contrast, IPS-PP and IPS-∆B2M-PP progenitors exhibited markedly lower viability, at 53.0 ± 22.8% and 57.5 ± 16.4%, respectively. For both progenitor lines, cells within clusters showed higher viability compared to isolated single cells, suggesting that, for these, cell–cell contacts provide a protective effect against encapsulation-related stress (Figure 4). Although the viability of IPS-PP and IPS-ΔB2M-PP cells was lower than that of the fibroblasts, a substantial fraction of progenitor cells remained viable at 24 h post-encapsulation, and no signs of complete cell death or necrosis were observed within the capsules. Since viable cells are required to elicit an immune response, this viability level was sufficient for the subsequent immunological experiments, although optimization of encapsulation conditions may be required to improve the survival of more sensitive cell lines.

### 3.4. Biocompatibility Assessment of Microcapsules

#### 3.4.1. In Vitro Biocompatibility by Assessment of Rat Macrophage Response

**Figure 5:**
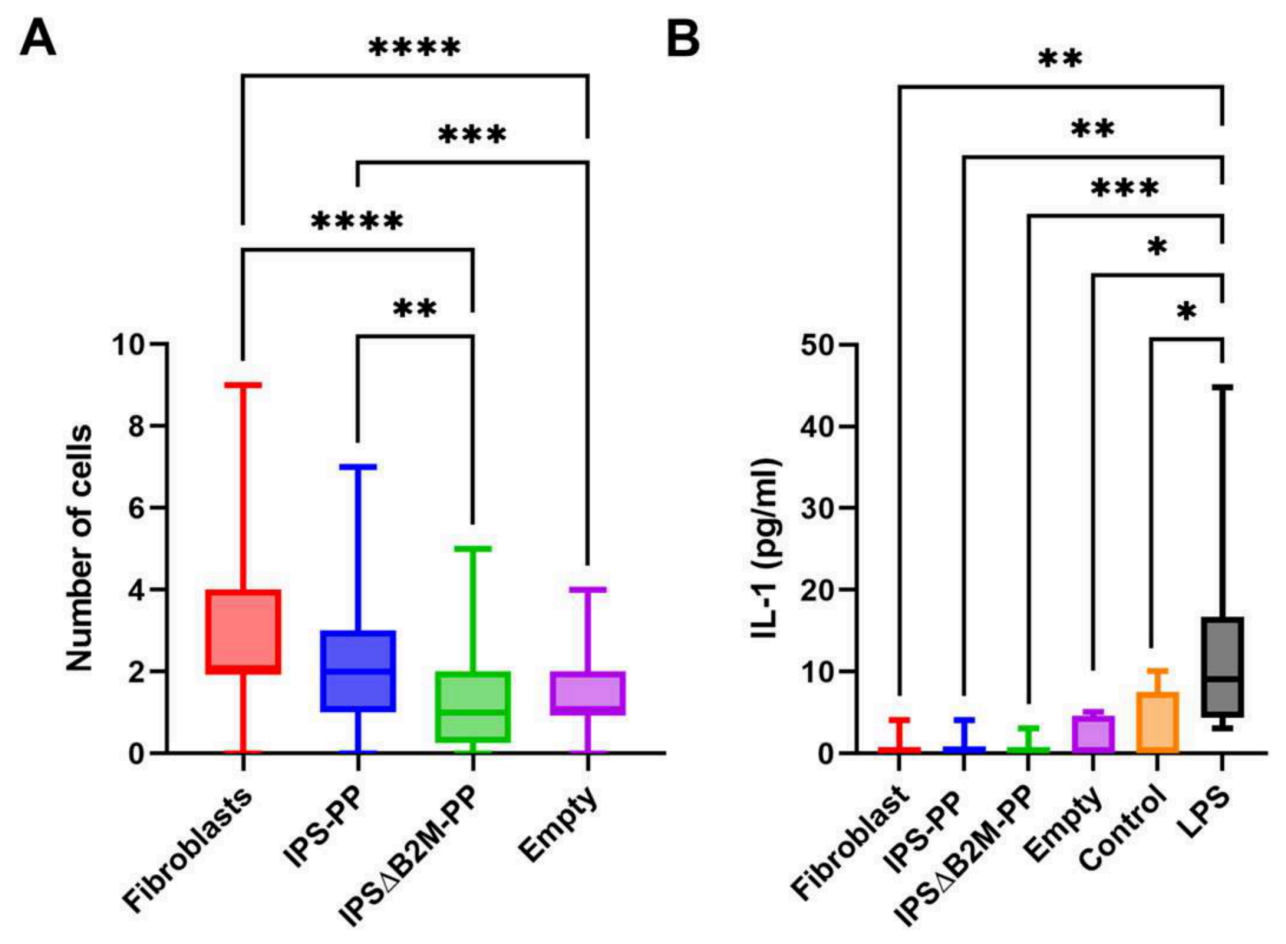
In vitro biocompatibility assessment of alginate microcapsules: macrophage adhesion and activation. (**A**) Quantitative analysis of macrophage adhesion to microcapsule surfaces after 24 h of co-culture. Empty microcapsules served as a negative control. Three independent batches with 50 capsules each (total = 150 capsules per group) were used. (**B**) Macrophage activation assessed by IL-1β secretion after 24-h co-incubation. Three independent batches with 3 technical replicates. Statistical test: (**A**,**B**) Kruskal–Wallis test followed by Dunn’s multiple comparisons test. Statistical significance: * *p* < 0.05, ** *p* < 0.01, *** *p* < 0.001, **** *p* < 0.0001.

Macrophage adhesion to the microcapsules was quantified after 24 h of co-culture. Empty microcapsules, used as a negative control, exhibited baseline adhesion of 1.28 ± 1.00 cells per capsule (Figure 5). Encapsulation of IPSs as well as fibroblasts significantly increased macrophage attachment, with mean values of 2.32 ± 1.88 and 2.76 ± 1.78 cells per capsule, respectively (*p* < 0.0001 vs. empty). By contrast, microcapsules containing IPS-∆B2M-PPs showed markedly less adhesion (1.47 ± 1.24 cells/capsule), this being not significantly different from the empty control (*p* = 0.8235) but significantly lower than for both the IPS-PP (*p* = 0.0002) and fibroblast-loaded capsules (*p* < 0.0001). These findings indicate that the absence of surface HLA-I on encapsulated cells correlates with a substantial reduction in macrophage attachment, bringing adhesive properties to a level comparable to that of the carrier material alone.

To evaluate the pro-inflammatory potential of the capsules, IL-1β secretion was measured in the culture supernatants after 24 h. The supernatants from LPS-stimulated macrophages served as a positive control and elicited a robust IL-1β response (13.27 ± 13.66 pg/mL), significantly higher than all other groups (*p* < 0.05). Across all experimental conditions, including empty capsules and those loaded with any cell type, the IL-1β levels remained consistently low, ranging from 0.39 ± 1.10 (IPS-∆B2M) to 1.86 ± 2.52 (empty) pg/mL. No significant differences were observed between any of the capsule-containing groups and the untreated control (3.04 ± 4.29 pg/mL; *p* > 0.05 for all pairwise comparisons).

Collectively, these data demonstrate that neither the alginate microcapsules nor their combination with IPS-PPs or fibroblasts elicits a pro-inflammatory IL-1β response. Furthermore, the use of B2M-knockout cells effectively minimizes macrophage adhesion, suggesting enhanced biocompatibility and reduced immunogenicity of the encapsulated grafts.

#### 3.4.2. In Vivo Biocompatibility After Transplantation in Rats

For in vivo biocompatibility assessment, encapsulated pancreatic progenitors (IPS-PPs and IPS-∆B2M-PPs) were transplanted into rat omentum. Encapsulated human fibroblasts served as controls. Tissue reactions were analyzed at 2 weeks and 1 month post-transplantation.

**Figure 6:**
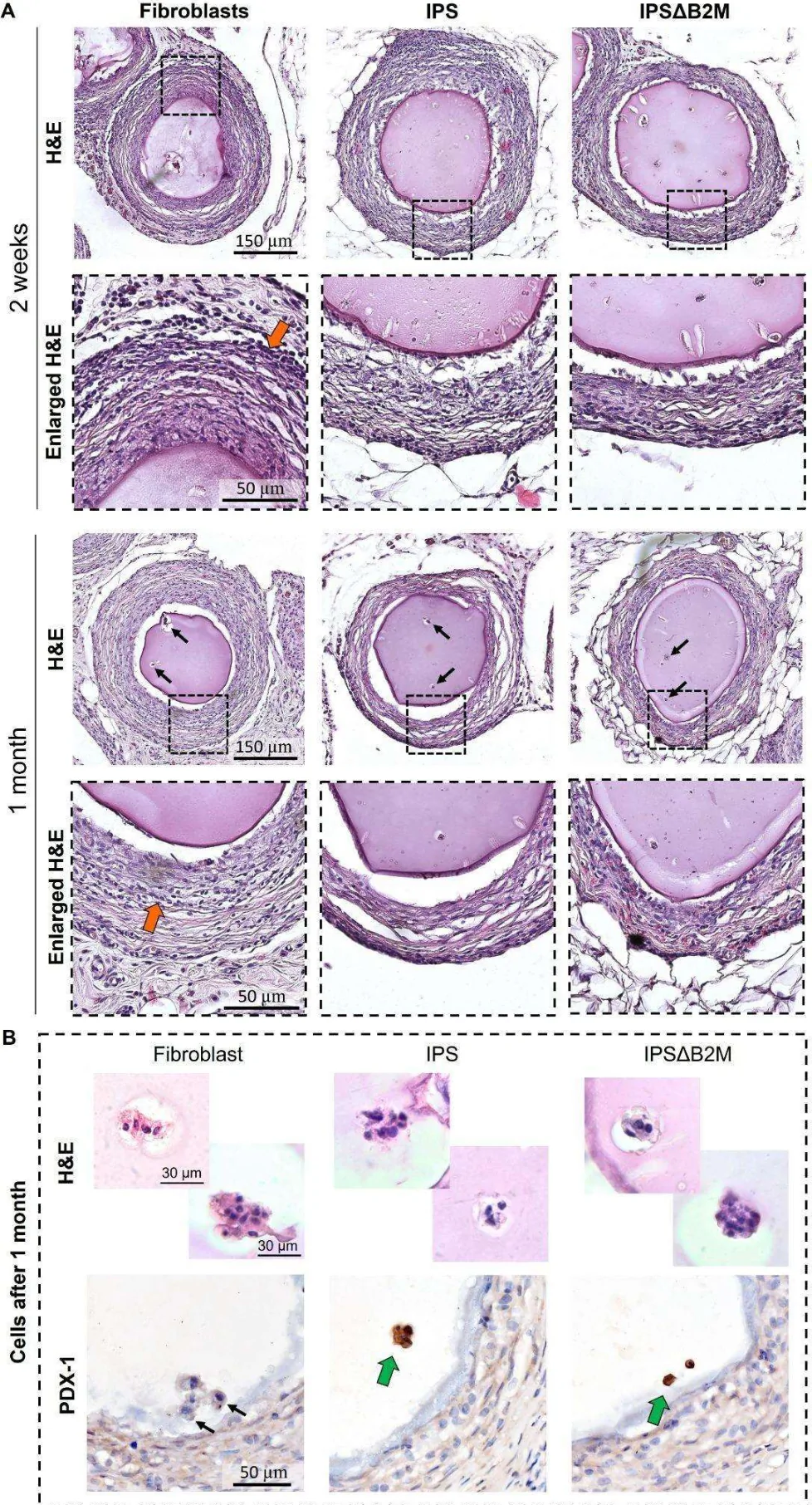
Representative histological images of the tissue response to microcapsules implanted into the omentum and morphometric analysis of the tissue reaction. (**A**) Hematoxylin and eosin (H&E) staining of omental tissue around implanted microcapsules. (**B**) Histological and IHC images of viable cells enclosed within microcapsules at 1 month after omental implantation. PDX-1 is expressed only in living pancreatic progenitor cells. Black arrows indicate groups and single viable cells within the microcapsules with no signs of apoptosis or necrosis; orange arrows indicate lymphocytic inflammatory infiltrate; green arrows indicate PDX-1-positive cells; dotted squares denote magnified regions. Three independent batches with 3 animals per time point (2 weeks and 1 month), giving a total of 18 animals across three experimental groups.

Histological examination revealed two distinct zones around the microcapsules regardless of the cell type within: an inner myofibroblastic zone with immature collagen, and an outer zone containing mature collagen, capillaries, and lymphocytic infiltrate. The immunohistochemical and morphometric analysis of the tissue responses to the microcapsules is provided in the Supplementary Materials (Figure S5). Importantly, no giant multinucleated foreign-body cells were detected at any time point, and macrophage staining (Iba-1) was negative on the capsule surface. Capsule integrity was largely preserved, with only 8–21% damaged capsules, as confirmed by the preserved nuclear architecture and the absence of morphological signs of apoptosis or necrosis. Viable cell conglomerates were observed inside some capsules at 1 month, corroborated by the preserved nuclear architecture and the absence of morphological signs of apoptosis or necrosis (Figure 6B).

The inflammatory and fibrotic response depended on the encapsulated cell type (Figure S5A). Fibroblast-containing capsules induced the most pronounced reaction, with the thickest fibrous capsules and the highest lymphocyte density. By contrast, IPS-PP capsules elicited a milder and looser fibrotic response with moderate lymphocytic infiltration. The lowest reactivity was observed around IPS-∆B2M-PP capsules, which consistently showed the thinnest myofibroblastic zone, minimal lymphocytic infiltrate, and no signs of capsule deformation, even at 1 month (Figure 6A).

### 3.5. Human Immune Cell Response

#### 3.5.1. Human NK Cell Allogeneic Response to Encapsulated Progenitors

To evaluate the allogeneic response of human NK cells to encapsulated and non-encapsulated pancreatic progenitors, we used the classical degranulation assay based on CD107a expression and compared the responses to non-edited pancreatic progenitors (IPS-PPs) without encapsulation in 2D culture to pancreatic progenitors with B2M deletion (IPS-∆B2M-PPs) without encapsulation in 2D culture, to encapsulated IPS-PPs, and to encapsulated IPS-∆B2M-PPs. Peripheral blood mononuclear cells (PBMCs) from six healthy donors were co-cultured with the encapsulated and non-encapsulated pancreatic progenitors for 4 h, and the percentage of degranulated CD56^+^CD107a^+^ cells was assessed. The erythromyeloid line K562 served as a positive control for NK activation, and unstimulated PBMCs served as a negative control. To account for donor-to-donor variability, the obtained values for each donor were normalized to the positive control (response to K562 set as 100%). Human fibroblasts cultured in monolayer and encapsulated human fibroblasts were used as control cells with high B2M expression levels.

**Figure 7:**
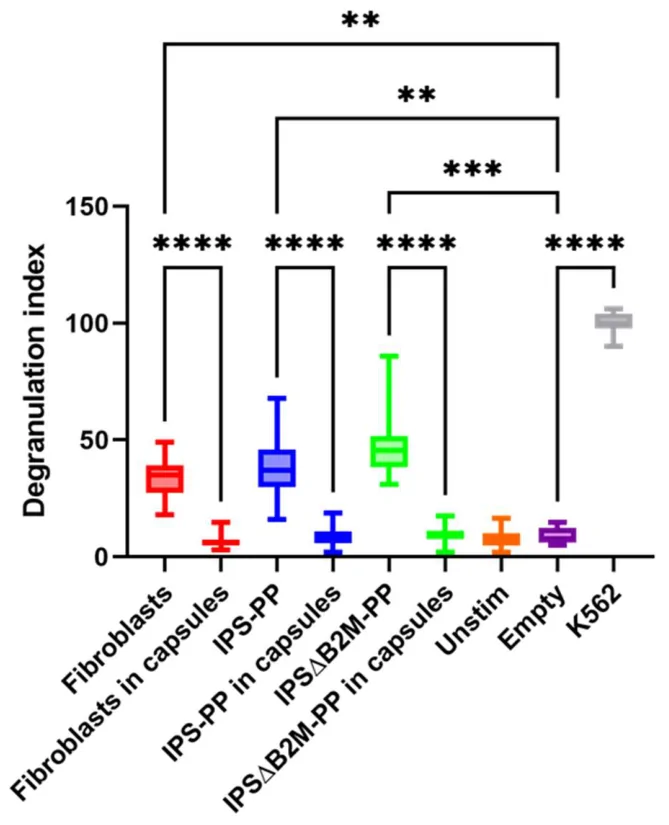
Degranulation index of NK cells against encapsulated and non-encapsulated cells. Empty capsules were used as a control. Six donors and 2 independent encapsulations (for each donor, each in triplicate) were used. Statistical test: Kruskal–Wallis test followed by Dunn’s multiple comparisons test. Statistical significance: ** *p* < 0.01, *** *p* < 0.001, **** *p* < 0.0001.

The NK cells demonstrated pronounced degranulation in the presence of all non-encapsulated cultures upon direct contact with the target cells in 2D culture (both fibroblasts and pancreatic progenitors) (Figure 7). The response level to fibroblasts was 34 ± 7%, to IPS-PPs the response was 37 ± 14% of the K562 level (*p* < 0.05 relative to the negative control), and to IPS-∆B2M-PPs it was 48 ± 14%. Notably, the IPS-PPs induced significantly less degranulation compared to the IPS-∆B2M-PPs (*p* = 0.03).

A fundamentally different pattern was observed when testing the encapsulated forms. Neither fibroblasts in capsules, encapsulated IPS-PPs, encapsulated IPS-∆B2M-PPs, nor empty capsules induced a significant increase in the proportion of degranulated NK cells compared to the unstimulated control: the corresponding values were 6 ± 3%, 8 ± 4%, 9 ± 4%, and 8 ± 3% (*p* > 0.05 in all cases) (Figure 7). Thus, encapsulation completely prevented NK cell activation regardless of the nature of the encapsulated cells, including under conditions of absent HLA-I molecules (B2M knockout), which in monolayer culture, as expected, did not reduce, but rather enhanced degranulation through the “missing-self” mechanism.

#### 3.5.2. Human T-Cell Allogeneic Response to Encapsulated Progenitors

**Figure 8:**
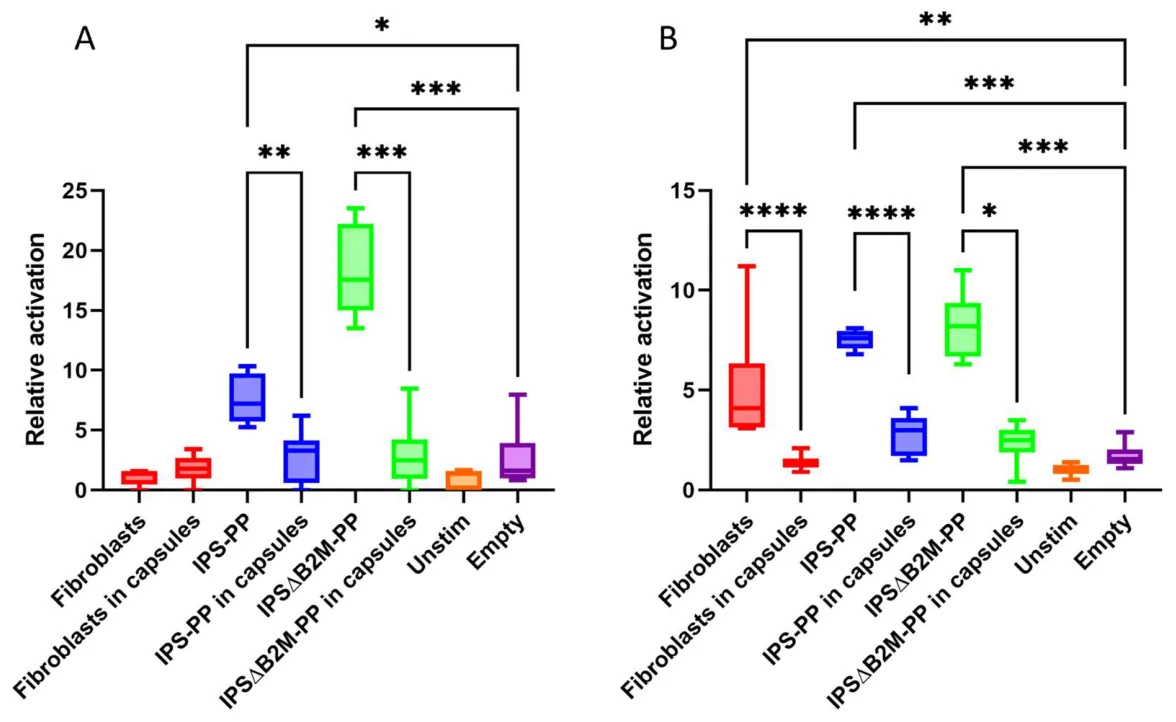
(**A**) Relative in vitro T-cell activity against encapsulated and non-encapsulated cells by assessing % of CD3^+^CD69^+^CD25^+^ cells at 24 h timepoint (early activation). Two donors and 2 independent encapsulations (for each donor, each in triplicate) were used. Statistical test: One-way ANOVA followed by Dunnett’s T3 multiple comparisons test. (**B**) Relative in vitro activation of T-cells against encapsulated and non-encapsulated cells by assessing % of CD3^+^CD69^+^ cells at 7-day timepoint (late activation). Empty capsules were used as a control. Four donors and 2 independent encapsulations (for each donor, each in triplicate) were used. Statistical test: Kruskal–Wallis test followed by Dunn’s multiple comparisons test. Statistical significance: * *p* < 0.05, ** *p* < 0.01, *** *p* < 0.001, **** *p* < 0.0001.

T-cell responses to encapsulated and non-encapsulated pancreatic progenitors were assessed by co-culturing them with PBMCs from healthy donors for 24 h and 7 days. For the 24-h stimulation, the immune response was evaluated by CD69 and CD25 expression on the T-cells. The obtained values were normalized to the percentage of activated cells in unstimulated PBMCs. All encapsulated cell variants, as well as monolayer fibroblasts, elicited T-cell responses at background levels (≤1 times the unstimulated control; *p* > 0.05) (Figure 8). The only targets that induced notable activation were non-encapsulated IPS-PPs. The response to IPS-PPs averaged 7.5 times the unstimulated control (*p* < 0.05), while the response to IPS-∆B2M-PPs was approximately twice as high, at 17 times the unstimulated control (*p* < 0.05). Thus, under 24-h stimulation, only non-encapsulated pancreatic progenitors in direct contact with immune cells triggered T-cell activation, with the absence of HLA-I (B2M knockout) significantly enhancing the response. Fibroblasts under the same conditions were non-immunogenic.

PBMCs from four donors were co-cultured with the same target types for 7 days, activation being assessed by CD69 expression on CD3^+^ T-cells (CD3^+^CD69^+^). The data were normalized to the unstimulated control (each point derived from two independent encapsulations in three replicates). The level in unstimulated PBMCs was set as 1-fold activation (baseline). Empty capsules and encapsulated fibroblasts did not significantly increase this proportion (1.4 ± 0.5 times and 1.4 ± 0.5 times, respectively; *p* > 0.05). Non-encapsulated fibroblasts induced significant activation, reaching 3.6 ± 1.6 times (*p* < 0.05), which contrasts with the near absence of a response to fibroblasts in the 24-h assay and indicates that fibroblasts require a longer time to trigger a T-cell response. Non-encapsulated pancreatic progenitors remained the most potent activators, with activation levels of 6.8 ± 0.6 times for the IPS-PPs and 7.4 ± 1.3 times for the IPS-∆B2M-PPs (both with *p* < 0.001 versus the unstimulated control). Encapsulation significantly reduced but did not completely abolish T-cell activation: encapsulated IPS-PPs yielded it at 2.7 ± 1.1 times, and encapsulated IPS-∆B2M-PPs yielded 2.5 ± 0.6 times. These levels were significantly higher than for the unstimulated cells (*p* < 0.05) but significantly lower than for the corresponding non-encapsulated cultures (*p* < 0.05). No major differences were observed between encapsulated IPS-PPs with normal HLA-I and those with B2M knockout.

Thus, in contrast to the NK-cell response, which was almost completely blocked by encapsulation, T-cells exhibited low-level activation upon prolonged co-culture with encapsulated pancreatic progenitors. Furthermore, the kinetic response to fibroblasts (no activation at 24 h, but activation at 7 days) suggests a delayed mechanism of T-cell engagement, possibly mediated by indirect antigen presentation.

## 4. Discussion

In recent years, the most promising direction for the treatment of type 1 diabetes mellitus (T1DM) has been cell replacement therapy based on IPSC-derived β-cells. The use of induced pluripotent stem cells (IPSCs) allows for an unlimited supply of mature β-cells, thereby addressing the shortage of donor organs. Unlike exogenous insulin therapy, transplantation of functional β-cells can restore physiological insulin secretion, eliminating the root cause of absolute insulin deficiency. A recent clinical study demonstrated that transplantation of stem cell-derived islet cells provides sustained C-peptide secretion and, in some patients, complete independence from exogenous insulin [17]. However, the major challenge for the long-term efficacy of cell therapy remains immune rejection of the graft. In allogeneic transplantation, IPSC derivatives are recognized by the patient’s immune system as foreign cells, which can lead to their immune rejection. Consequently, new strategies to overcome graft rejection are being actively explored. One potential solution is genetic modification of the donor cells to alter their expression of molecules involved in transplant recognition by the recipient’s immune system cells. For example, hypoimmunogenic ∆B2M-β-cells can function in the recipient’s body while remaining less visible to the immune system [18].

Other approaches to overcome immune rejection are also being investigated in parallel, including the use of autologous IPSCs, which could theoretically reduce the risk of alloimmune rejection, and cell encapsulation in semipermeable hydrogel matrices. However, autologous IPSC therapy faces limitations such as its extremely high cost and lengthy manufacturing times, since the cell product must be generated individually for each patient, whereas allogeneic cell lines are far better suited for standardized and scalable production [19]. In the present study, we combined genetic modification via B2M knockout to reduce triggering of the immune response, with alginate encapsulation to minimize physical contact between transplanted cells and immune cells. Our study demonstrated that IPS cells with B2M knockout efficiently differentiated into pancreatic progenitors, and confirmed the absence of B2M expression by day 16 of differentiation, when compared to the non-knockout line. We did not observe any substantial differences in the percentage of pancreatic markers of the WT and B2M-knockout cells at this pancreatic progenitor induction stage even though comparison of the efficiency of differentiation was not the aim of the study. The next step was to encapsulate the obtained PP cells for further in vitro immunological tests and for assessing capsule biocompatibility and structural stability during in vivo studies.

Alginate encapsulation remains one of the most actively developed strategies for the immune protection of cell grafts in type 1 diabetes mellitus [20]. The creation of a semipermeable barrier around transplanted cells limits direct contact with effector immune cells while still allowing diffusion of oxygen, nutrients, and insulin [21]. However, the clinical application of encapsulated cells is limited by the foreign body reaction (FBR), which begins with plasma protein adsorption onto the capsule surface, followed by the recruitment and activation of macrophages, leading to fibrotic overgrowth, impaired mass exchange, and graft loss [22].

In our earlier experiments [8], we coated the alginate capsules with PMETAC to decrease cell adhesion. This did reduce fibrosis, but the capsules were still subject to degradation by macrophages, thereby losing their barrier function. Therefore, to stabilize the structure and reduce the local positive charge, NVP was added to the formulation. At one month post-transplantation, loss of capsule integrity was observed in 20% of the capsules. Damage or degradation of the capsule may expose encapsulated cells to the immune system, making them vulnerable to rejection through direct presentation of allogeneic HLA-I molecules. Application of B2M gene knockout in pancreatic progenitors eliminates surface expression of HLA-I and reduces the risk of immune recognition in the event of capsule rupture. However, the combination of alginate encapsulation and genetic modification of cells remains poorly studied, and the potential contribution of each strategy to immune protection, as well as their possible interactions, has not been systematically characterized.

Assessment of macrophage adhesion to the surface of microcapsules and their secretion of interleukin-1β (IL-1β) in vitro are reliable predictive markers of the early inflammatory response to biomaterials [22]. In our study, empty alginate microcapsules demonstrated excellent baseline inertness, resulting in minimal cell attachment and only basal IL-1β levels. This is fully consistent with the literature, according to which the use of highly purified and low-viscosity alginates minimizes the initial cascade of myeloid cell activation directly on the polymer carrier itself [23]. However, encapsulation of the IPS-PP cells significantly enhanced macrophage adhesion in vitro, confirming the phenomenon of antigen shedding and the free diffusion through the pores of the semipermeable membrane of chemokines and cell stress factors (DAMPs) that attract and activate the recipient’s macrophages [23]. We observed that while encapsulation of IPS-PP cells increased macrophage adhesion compared to empty capsules, adhesion to IPS-ΔB2M-PP-containing capsules was comparable to that of empty capsules (Figure 5A). Since macrophages do not express T-cell receptors and B2M knockout is actually designed to evade CD8^+^ T-cell recognition, the reduced macrophage adhesion to IPS-ΔB2M-PP capsules was unexpected. We propose two possible, non-mutually exclusive explanations, both of which remain to be tested experimentally. First, soluble HLA-I molecules released from the capsules may act as direct stimuli recognized by innate immune receptors on the macrophages; B2M deletion would reduce the number of such molecules released. Second, B2M deletion may alter the secretome of the progenitors, reducing the release of myeloid chemokines such as CCL2. Further studies, including secretome profiling and chemokine blockade, are required to distinguish between these possibilities.

In the in vivo assessment of capsule biocompatibility and the local tissue response to microcapsules implanted into the rat omentum, the formation of two distinct zones around the microcapsules was observed: The first zone consisted of myofibroblasts containing abundant immature collagen fibers and was in direct contact with the microcapsule. The second zone was represented by mature, circularly arranged collagen fibres and bundles that contained lymphocytic infiltrate, macrophages, and small blood capillaries (Figure S5). A notable finding was the absence of a dense macrophage layer and multinucleated foreign-body giant cells at the capsule surface. These features are commonly associated with persistent foreign-body responses to implanted biomaterials [24]. Their absence at the evaluated time point supports the favorable biocompatibility of the capsule surface. These findings indicate attenuation rather than complete absence of the foreign-body response.

The severity of the inflammatory response in the omentum and the characteristics of the developing capsules depended significantly on the type of encapsulated cells. The most intense reaction was observed around capsules containing fibroblasts, where the myofibroblastic zone thickness and lymphocyte infiltrate density were maximal. Only in this group did the thickness of the mature fibrotic capsule exceed 100 μm—a threshold value at which oxygen diffusion becomes limited, and which, according to the literature, leads to reduced functional activity of the encapsulated islet cells [24]. By contrast, capsules containing IPS-PP pancreatic cells and especially IPS-∆B2M-PPs induced significantly less pronounced fibrogenesis and inflammation. The presence of small blood vessels within the outer fibrotic zone may facilitate local oxygen and nutrient supply and could contribute to the preservation of encapsulated cells. We speculate that the absence of B2M may contribute to the reduced inflammatory and fibrotic response observed around IPS-ΔB2M-PP capsules, but the underlying mechanism was not directly investigated. Possible explanations include reduced release of HLA-I-derived antigens and altered secretion of pro-inflammatory factors by the B2M-knockout cells.

In vitro evaluation of the response of human immune cells revealed a fundamentally different sensitivity of NK and T-cells to encapsulation of pancreatic progenitors. NK cells showed minimal degranulation in the presence of any of the encapsulated targets, whereas T-cells, under prolonged 7-day co-culture, retained low-level activation, although significantly lower than the activation by non-encapsulated cells. We hypothesize that this difference may be explained by several complementary mechanisms, although none of these mechanisms was directly tested in our study.

First, mechanical damage to some capsules during culture cannot be ruled out. Even a minor fraction of progenitors released from such damaged capsules could potentially come into direct contact with immune cells and trigger a T-cell response [25,26]. In contrast to NK cells, for which immediate contact with surface ligands of “stress” and “missing-self” is critical, T lymphocytes are able to respond to antigens presented either directly or indirectly—after processing by antigen-presenting cells [27,28]. Thus, single “escaped” cells could potentially initiate T-cell activation accumulating over 7 days but be insufficient for a detectable NK signal.

Second, T-cells—particularly subsets of effector memory CD8^+^ T-cells—are capable of TCR-independent killing through innate immune receptors, notably NKG2D [29]. It is known that IPSC derivatives are characterized by their upregulated expression of stress-inducible molecules (MICA/B, ULBP, etc.) [30] that serve as ligands for NKG2D. Furthermore, non-encapsulated, IPS-∆B2M-PP cells that are completely devoid of classical HLA-I molecules induced early T-cell activation twice as strongly as unmodified IPS-PPs. We therefore hypothesize that this reflects the concept of “missing-self” combined with stress signaling: the absence of inhibitory HLA-I while stress ligands are retained makes the target more susceptible to TCR-independent recognition by T-cells [31]. While encapsulation likely physically limits immune cell access to progenitors, it does not completely eliminate the possibility of contact with escaped cells or the presentation of their antigens by dendritic cells, and this could explain the residual response to encapsulated pancreatic cells. At the same time, the fact that the response remained at a level substantially lower than without encapsulation indicates that the physical barrier of a capsule is the primary protective factor.

Third, the fact that encapsulated pancreatic progenitors, even with B2M deletion, induced only minimal NK cell degranulation suggests that NK cells may have restricted access to the target surface in intact capsules. Even if individual cells escape from the capsules, their number appears insufficient to evoke measurable NK degranulation in the 4-h assay, the sensitivity of which is lower than that of the T-cell protocol with prolonged stimulation. This is consistent with the notion that NK-cell activation requires either direct contact or a high concentration of soluble stress ligands, and the likelihood that encapsulation substantially reduces both.

Overall, the present study demonstrates the feasibility of combining alginate encapsulation with B2M knockout in iPSC-derived pancreatic progenitors and highlights the distinct effects of these strategies on immune cell interactions. Under the conditions tested, encapsulation provided the predominant protection against NK-cell degranulation, while B2M deletion did not confer any additional reduction in T-cell activation in the presence of intact capsules. The persistence of residual T-cell activation indicates that the current approach does not provide complete immune protection. The potential advantage of B2M deletion in the event of compromised capsule integrity remains biologically plausible but was not directly evaluated in this study.

Our in vivo experiments were focused on capsule biocompatibility, local tissue response, and structural stability rather than immune recognition of the encapsulated cells. This model was therefore not intended fully to recapitulate human allogeneic immune recognition, and the small sample size (n = 3 animals per group per time point) limits the statistical power and generalizability of these observations. Other aspects we did not specifically investigate include detailed characterization of the fate and phenotypes of the transplanted cells; assessment of their differentiation status, of their endocrine functionality or of any potential undesirable changes As the focus was on immune interactions and biocompatibility rather than therapeutic efficacy, the study used pancreatic progenitors rather than mature insulin-producing β-cells, and functional graft efficacy was not evaluated in a diabetic model.

Further optimization of both biomaterial properties and genetic engineering, including approaches such as CIITA knockout or CD47 overexpression, may therefore be required to achieve more robust immune protection. Additional studies in humanized models and under conditions of compromised capsule integrity will be necessary to determine whether combining the above strategies provides additional protection against human immune responses in vivo.

Thus, although the combination of encapsulation with B2M gene knockout significantly reduced immune cell interactions in vitro and attenuated the foreign body response in vivo, the residual T cell activation indicates that the current techniques still provide somewhat incomplete immune protection. However, the multilayered approach used in the present study provides a rational framework for further development of immune-protected cell replacement therapies for diabetes.

## Data Availability

The original contributions presented in this study are included in the article/Supplementary Material. Further inquiries can be directed to the corresponding author(s).

## Supplementary Materials

The following supporting information can be downloaded at: https://www.mdpi.com/article/10.3390/cells15211942/s1, Figure S1. Flow cytometry analysis and gating strategy for T-cell activity at 24 h timepoint; Figure S2. Flow cytometry analysis and gating strategy for T-cell activity at 7 day timepoint; Figure S3. Flow cytometry analysis and gating strategy for NK cells; Table S1. Primers used for RT-PCR; Table S2. Stability of Microcapsules. Dimensional characteristics of microcapsules containing fibroblasts, IPS-PP, and IPSΔB2M-PP cells after 1 h and 1 day of incubation in HBBS, complete culture medium, and FBS. Data include sample size (n), mean diameter, standard deviation (SD), minimum and maximum diameter, coefficient of variation (CV), and polydispersity index (PDI).; Supplementary Text S1. Osmotic stability test; Figure S4. Osmotic stability of alginate microcapsules. Microcapsules were incubated in distilled water (H_2_O) for 24 h to induce hypotonic swelling, then returned to HBSS and measured at 15 min, 24 h, and 48 h. Representative bright-field images of microcapsules at each time point. Data are presented as mean ± SD; n = 100–150 capsules per condition. **** *p* < 0.0001, ** *p* < 0.01, * *p* < 0.05, ns—not significant; Figure S5. (a) Morphometric analysis of the tissue response to microcapsules: (a) immunohistochemical analysis of the tissue response to microcapsules, demonstrating a clearly delineated zone of myofibroblasts (α-SMA) around the microcapsule and macrophages localized within the fibrotic area (Iba-1); (b) Masson’s trichrome staining showing the formation of a fibrotic zone around the microcapsules; (c) assessment of myofibroblastic zone thickness and fibrotic capsule thickness around the microcapsules. MfZ, myofibroblastic zone; Mφ, macrophage; Fb, fibrosis.

## Abbreviations

The following abbreviations are used in this manuscript:

IPSCs	induced pluripotent stem cells
B2M	β2-microglobulin
DE	definitive endoderm
PP	pancreatic progenitor
T1DM	type 1 diabetes mellitus
DM	diabetes mellitus
NK cells	natural killer cells
IL-1β	interleukin-1β
PBMCs	peripheral mononuclear blood cells
HLA	human leukocyte antigen
FBR	foreign body response
