# Supplementary material for: Combined B2M Knockout and Alginate Encapsulation Reduces Immune Rejection of iPSC-Derived Pancreatic Progenitors
Source: Cells. 2026 Oct 26;15(21):1942. doi: 10.3390/cells15211942 (PMC13761956; doi:10.3390/cells15211942)
Supplement: Supplementary file 1 [file cells-15-01942-s001.zip › cells-4566810-supplementary.pdf]

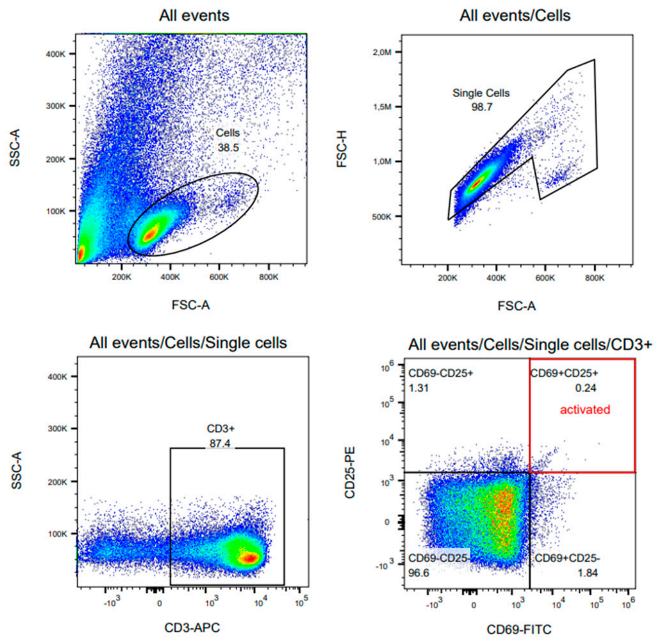

Figure S1. Flow cytometry analysis and gating strategy for T-cell activity at 24h timepoint

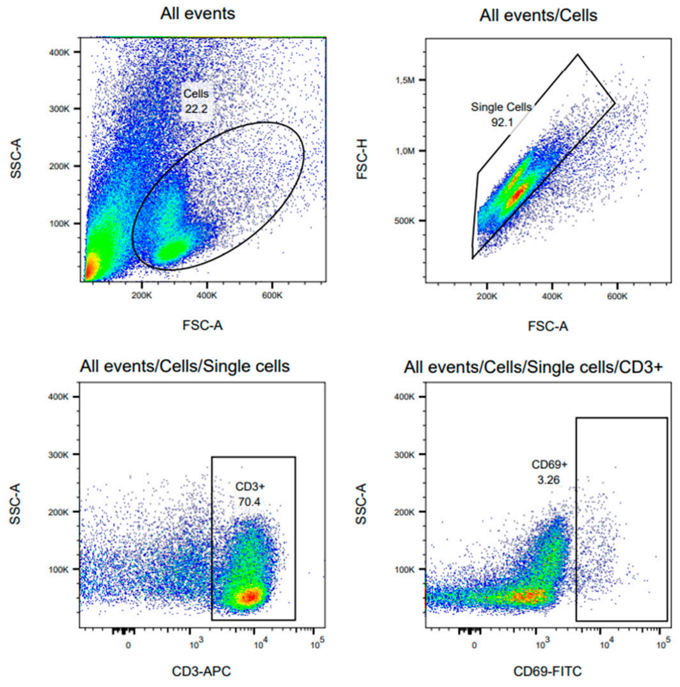

Figure S2. Flow cytometry analysis and gating strategy for T-cell activity at 7 day timepoint

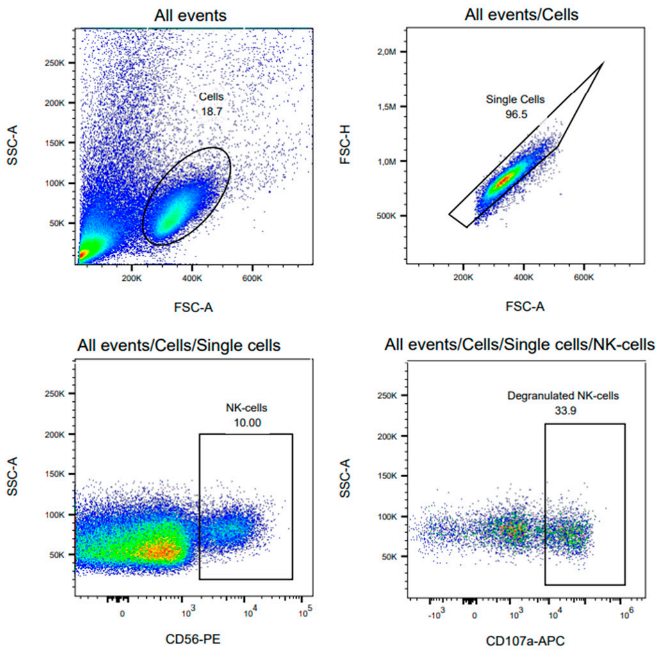

Figure S3. Flow cytometry analysis and gating strategy for NK cells

Table S1. Primers used for RT-PCR

Gene name	Sequence forward	Sequence reverse
PDX1	CGTCCGCTTGTTCTCCTC	CCTTTCCCATGGATGAAGTC
INS	CAATGCCACGCTTCTGC	TTCTACACACCCAAGACCCG
ISL1	TCACGAAGTCGTTCTTGCTG	CATGCTTTGTTAGGGATGGG
SST	TGGGTTCAGACAGCAGCTC	CCCAGACTCCGTCAGTTTCT
GCG	AGCTGCCTTGTACCAGCATT	TGCTCTCTCTTCACCTGCTCT

Table S2. Stability of Microcapsules. Dimensional characteristics of microcapsules containing fibroblasts, IPS-PP, and IPSΔB2M-PP cells after 1 hour and 1 day of incubation in HBBS, complete culture medium, and FBS. Data include sample size (n), mean diameter, standard deviation (SD), minimum and maximum diameter, coefficient of variation (CV), and polydispersity index (PDI).

Cell Type	Condition	Time Point	n	Mean Diameter (μm)	SD (μm)	Min (μm)	Max (μm)	PDI
Fibroblasts	HBBS	1 hour	150	263.7	62.4	94.8	435.8	0.056
Fibroblasts	HBBS	1 day	150	253.8	66.9	103.8	434.3	0.070
Fibroblasts	Culture medium	1 hour	150	268.3	66.7	94.4	506.9	0.062
Fibroblasts	Culture medium	1 day	150	262.1	64.5	108.1	486.4	0.061
Fibroblasts	FBS	1 hour	150	248.5	60.9	129.6	497.9	0.060
Fibroblasts	FBS	1 day	150	246.7	57.7	114.4	425.1	0.055
IPS-PP	HBBS	1 hour	150	261.6	63.7	88.6	338.4	0.059
IPS-PP	HBBS	1 day	150	255.9	61.3	122.6	369.7	0.057
IPS-PP	Culture medium	1 hour	150	268.1	55.3	120.2	363.4	0.043
IPS-PP	Culture medium	1 day	150	253.9	58.4	114.1	396.2	0.053
IPS-PP	FBS	1 hour	150	258.0	61.8	112.8	347.7	0.057
IPS-PP	FBS	1 day	150	253.6	57.8	109.0	335.5	0.052
IPSΔB2M-PP	HBBS	1 hour	150	261.4	58.2	104.4	356.5	0.050
IPSΔB2M-PP	HBBS	1 day	150	265.3	53.0	96.6	352.5	0.040
IPSΔB2M-PP	Culture medium	1 hour	150	271.9	51.6	133.2	369.4	0.036
IPSΔB2M-PP	Culture medium	1 day	150	274.5	50.5	129.6	342.5	0.034
IPSΔB2M-PP	FBS	1 hour	150	274.4	51.3	117.6	338.5	0.035
IPSΔB2M-PP	FBS	1 day	150	263.7	53.8	114.6	371.2	0.042

Supplementary Text S1. Osmotic stability test

Osmotic stability of the microcapsules was assessed by exposure to a hypotonic solution, which induces shell stretching and serves as a standard model of mechanical loading that reveals latent defects in the capsule material. Although such abrupt osmotic gradients do not occur in vivo, this test has been validated as a predictive tool for in vivo capsule performance: Van Raamsdonk and Chang (2001) demonstrated a direct correlation between in vitro osmotic resistance and long-term in vivo survival of implanted capsules. The test also allows evaluation of the viscoelastic properties of the shell, including elasticity and plastic deformation (Verheyen et al., 2019).

Microcapsules were incubated in distilled water (H_2O) for 24 h and then returned to HBSS, with diameter measurements taken at 15 min, 24 h, and 48 h after the return. Exposure to H_2O caused a pronounced but reversible increase in mean diameter (319.9 ± 57.7 μm vs. 253.1 ± 68.7 μm in HBSS at 24 h; $p < 0.0001$, Dunn's test), consistent with elastic swelling of the polymer network. After transfer back to HBSS, the capsules rapidly recovered their size: mean diameter decreased to 288.0 ± 64.4 μm at 15 min, 265.2 ± 53.4 μm at 24 h, and 270.7 ± 55.3 μm at 48 h, with no significant difference from the HBSS control at 48 h ($p > 0.9999$). Importantly, no capsule rupture, fragmentation, or loss of morphological integrity was observed at any time point (Figure S2). These results confirm that the microcapsules possess sufficient elasticity and structural resilience to withstand osmotic stress without irreversible deformation, supporting their suitability for transplantation.

Van Raamsdonk J. M., Chang P. L. Osmotic pressure test: A simple, quantitative method to assess the mechanical stability of alginate microcapsules // Journal of Biomedical Materials Research. – 2001. – Vol. 54. – № 2. – P. 264- 271.

Verheyen C. A., Morales L., Sussman J. et al. Characterization of polyethylene glycol--reinforced alginate microcapsules for mechanically stable cell immunoisolation / // Macromolecular Materials and Engineering. – 2019. – Vol. 304. – № 4. – P. 1800679.

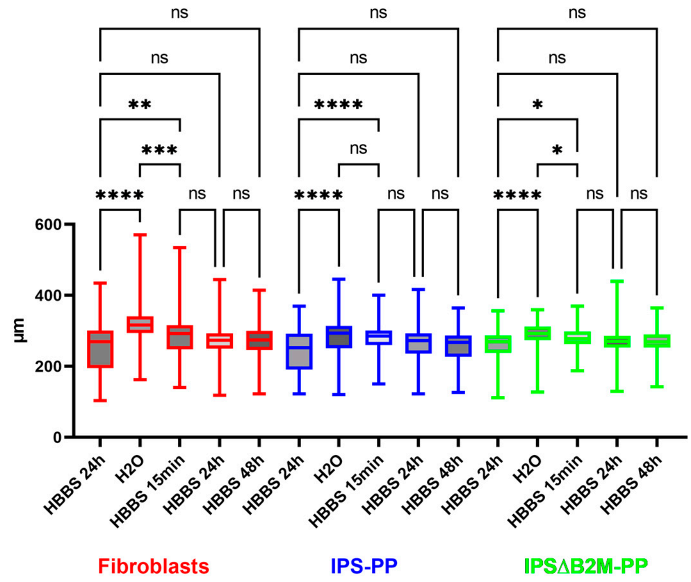

Figure S4. Osmotic stability of alginate microcapsules. Microcapsules were incubated in distilled water (H_2O) for 24 h to induce hypotonic swelling, then returned to HBSS and measured at 15 min, 24 h, and 48 h. Representative bright-field images of microcapsules at each time point. Data are presented as mean ± SD; n = 100–150 capsules per condition. ****$p < 0.0001$, **$p < 0.01$, *$p < 0.05$, ns — not significant

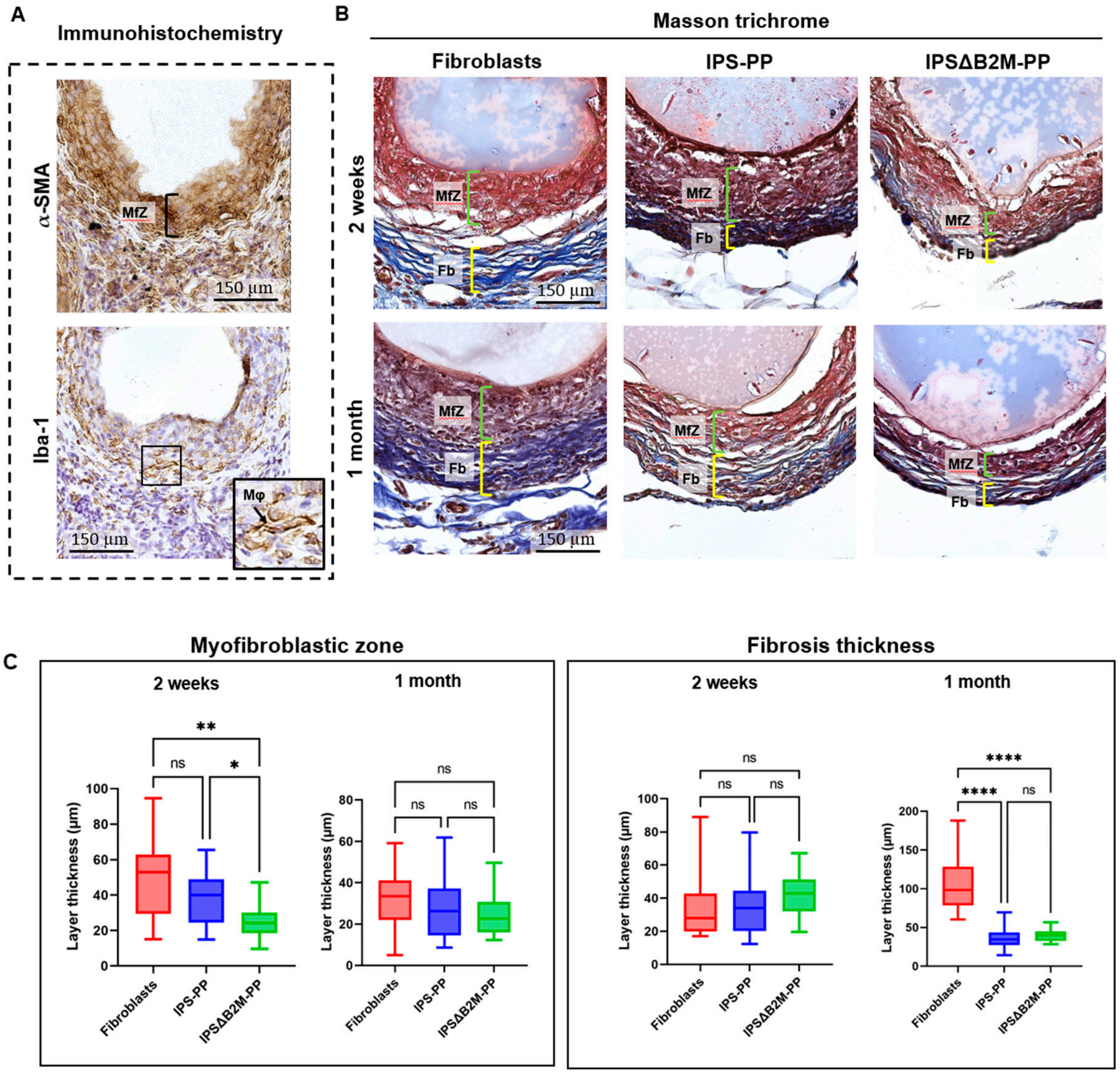

Figure S5. (a) Morphometric analysis of the tissue response to microcapsules: (a) immunohistochemical analysis of the tissue response to microcapsules, demonstrating a clearly delineated zone of myofibroblasts (α-SMA) around the microcapsule and macrophages localized within the fibrotic area (Iba-1); (b) Masson's trichrome staining showing the formation of a fibrotic zone around the microcapsules; (c) assessment of myofibroblastic zone thickness and fibrotic capsule thickness around the microcapsules. MfZ, myofibroblastic zone; Mφ, macrophage; Fb, fibrosis.
